# Healthy food diversity and the risk of major chronic diseases in the EPIC-Potsdam study

**DOI:** 10.1038/s41598-024-78287-5

**Published:** 2024-11-19

**Authors:** Daniela V. Nickel, Franziska Jannasch, Elif Inan-Eroglu, Olga Kuxhaus, Matthias B. Schulze

**Affiliations:** 1 Department of Molecular Epidemiology, German Institute of Human Nutrition Potsdam-Rehbruecke, 14558 Nuthetal, Germany; 2 NutriAct Competence Cluster Nutrition Research Berlin-Potsdam, 14558 Nuthetal, Germany; 3 German Center for Diabetes Research (DZD), 85764 Neuherberg, Germany; 4 Institute of Nutritional Science, University of Potsdam, 14558 Nuthetal, Germany; 5 Berlin School of Public Health, Charité-Universitätsmedizin Berlin, 10117 Berlin, Germany

**Keywords:** Type 2 diabetes, Myocardial infarction, Stroke, Risk factors

## Abstract

Practicing a diverse diet may reduce chronic disease risk, but clear evidence is scarce and previous diet diversity measures rarely captured diet quality. We investigated the effect of the Healthy Food Diversity (HFD)-Index on incident type 2 diabetes (T2D), myocardial infarction (MI) and stroke among a middle-aged German population. The EPIC-Potsdam study recruited 27,548 participants from 1994 to 1998. Semiquantitative food frequency questionnaire was used to calculate the HFD-Index. Longitudinal associations of HFD-Index and verified incident diseases were investigated by multiple-adjusted Cox proportional hazards regression models. Among 26,591 participants (mean age 50.5 years, 60% women), 1537, 376 and 412 developed T2D, MI and stroke, respectively, over an average follow-up of 10.6 years. There was no association between HFD-Index and incident T2D or MI. Higher compared to lower HFD-Index was inversely associated with incident stroke in men [HR (95% CI): 0.80 (0.70, 0.92)], but positively associated with incident stroke in women [1.20 (1.01, 1.42)]. Although there was no clear association between HFD-Index and T2D or MI incidence, we found a beneficial association in men and a harmful association in women for incident stroke. We emphasised the need for further investigations on combining diet diversity and diet quality in relation to health outcomes.

## Introduction

Major chronic diseases including myocardial infarction (MI), stroke and type 2 diabetes (T2D) were responsible for the majority of all deaths worldwide in 2019^1^. In Germany, even 91% of all deaths in 2016 were traced to major chronic diseases^2^. Among risk factors, dietary behaviour is considered a key modifiable determinant of the development of cardiovascular diseases (CVDs) and T2D^3^. Therefore, national and international policies have been implemented to engage individuals in eating a healthy and diverse diet^3^. Many international dietary recommendations emphasise the role of diet diversity to support the consumption of a large variety of foods and to promote adequate intake of essential nutrients^4–8^. Additionally, diet diversity may have a positive influence on health outcomes such as a reduced risk of T2D^9,10^. However, the evidence supporting a beneficial association between diet diversity and health outcomes is scarce and study results are inconsistent^11–13^. Although being recommended in dietary guidelines, it is unclear how diet diversity is conceptualised and measured, and how this could be transferred into healthy eating behaviours^11–13^.

Diverse diets differ in diet quality and most diet diversity indices are hardly able to distinguish between diversity within healthy and unhealthy foods^11^. Since high diet quality assessed by well-established diet quality scores was inversely associated with chronic disease risk^14^, the association between diet diversity and health outcomes may depend on the healthiness of the diet^13^. Therefore, to investigate diet diversity within a health-oriented, high-quality diet, Drescher et al*.* developed the Healthy Food Diversity (HFD)-Index to simultaneously consider diet diversity defined as the number of consumed foods, proportionality defined by the distribution of food groups in the diet and diet quality in relation to the German dietary guidelines^15^. The HFD-Index was further adapted to the 2010 Dietary Guidelines for Americans^16^. Based on energy-adjusted correlations with diet quality indicators such as nutrient supply, food groups and biochemical parameters, e.g. blood serum cholesterol and homocysteine, both the German and the US HFD-Index were considered as suitable for measuring healthy food diversity^15,16^.

While previous literature from the US and China revealed an inverse association of the HFD-Index with adiposity indicators, such as obesity and waist circumference, and metabolic syndrome^17–20^, limited studies between the HFD-Index and major chronic diseases yielded inconsistent results^21,22^. To our knowledge, the HFD-Index’s ability to longitudinally influence the risk of major chronic diseases has not been investigated before.

Therefore, the first aim of this study is to describe healthy food diversity measured by the HFD-Index among middle-aged German participants of the European Prospective Investigation into Cancer and Nutrition (EPIC) - Potsdam study at baseline. Second, we will investigate the association between the HFD-Index and the risk of major chronic diseases (T2D, MI, and stroke) in the EPIC-Potsdam cohort.

## Results

### General characteristics of the study population

Table 1 shows the descriptive characteristics of participants (N = 26,591). The mean age was 50.5 (SD: 9.0) years and 60% were females. They were mostly well-educated and in full-time occupation. Most of the participants were current alcohol drinkers and 51% were either current or former smokers. Men with higher HFD-Index were higher educated, and less likely drinking alcoholic beverages above the recommended upper limits or being current or former smokers compared to those with a lower HFD-Index. With regard to the mentioned characteristics, women showed smaller differences in the same direction across the groups of the HFD-Index. The mean body mass index (BMI) was 26.3 (SD: 4.3) kg/m^2^ and 49% of the population suffered from prevalent hypertension. Both men and women with higher adherence to the HFD-Index were more likely to have prevalent hypertension and hyperlipidaemia than those with lower adherence (Table 1).

**Table 1:** Descriptive characteristics across the tertiles of the Healthy Food Diversity (HFD)-Index among 10,551 men and 16,080 women from the EPIC-Potsdam study population at baseline.

Characteristic	Total	Men			Women		
		Tertile 1	Tertile 2	Tertile 3	Tertile 1	Tertile 2	Tertile 3
HFD-Index (range)	0.10–0.89	0.10–0.42	0.42–0.50	0.50–0.76	0.10–0.48	0.48–0.56	0.56–0.89
Number	26,591	3502	3504	3505	5359	5361	5360
Sociodemographic							
Age at recruitment (years)	50.5 (9.0)	51.6 (8.1)	52.9 (7.9)	53.2 (7.9)	48.4 (9.3)	49.5 (9.3)	49.5 (9.3)
Education (%): University degree or technical college	61.7	60.0	69.1	70.9	55.0	60.3	60.4
Occupation (%): Full-time	60.2	70.1	67.4	64.7	56.3	54.5	55.6
Lifestyle							
Lifetime alcohol pattern (%)							
Current, always below limit	53.9	33.9	36.4	40.9	64.5	64.7	65.5
Current, never heavy	29.6	41.3	43.8	38.6	21.6	22.9	21.7
Smoking status (%)							
Never smokers	48.7	28.5	31.3	32.1	55.9	60.2	58.2
Current or former smokers	51.4	71.5	68.7	67.9	44.1	39.8	41.8
Sports (hrs/wk; median (IQR))	4.5 (2.0, 8.5)	5.0 (2.0, 9.5)	5.5 (2.5, 10.0)	5.5 (2.5, 10.0)	4.0 (1.5, 7.5)	4.5 (2.0, 8.0)	4.5 (2.0, 8.0)
Metabolic conditions							
Body mass index (kg/m^2^)	26.3 (4.3)	26.8 (3.7)	27.0 (3.7)	27.3 (3.6)	25.7 (4.8)	25.8 (4.7)	25.9 (4.5)
Waist circumference (cm)	86.3 (13.0)	94.5 (10.3)	94.8 (10.1)	95.4 (10.0)	80.7 (11.9)	80.6 (11.5)	80.6 (11.3)
Baseline disease prevalence (%)							
Hypertension	48.8	54.9	57.9	63.5	40.2	42.7	43.7
Type 2 diabetes	5.4	5.1	8.2	8.8	3.7	4.6	4.1
Myocardial infarction	1.8	2.8	3.6	4.7	0.6	0.5	0.6
Stroke	1.0	1.3	1.3	1.6	0.7	0.7	0.8
Hyperlipidaemia*	27.3	30.0	32.5	38.5	21.5	23.0	25.0

Data are shown as mean (standard deviation) unless otherwise stated. hrs = hours; wk = week; IQR = interquartile range. *missing information on hyperlipidaemia in n = 150.

### Dietary characteristics of the study population

Descriptive statistics of dietary components across the three groups of the HFD-Index are presented for men and women in Table 2. The mean HFD-Index was 0.49 (SD: 0.10), being slightly higher in women compared to men [0.51 (0.09) versus 0.46 (0.09), respectively]. The distribution of the HFD-Index and its components is presented in Supplementary Fig. S1. The population had a high diet diversity expressed by the Berry-Index (Supplementary Fig. S1; Table 2), which was left-skewed and had a range of 0.42–0.99 and 0.35–0.98 in men and women, respectively. The Health Value was nearly normally distributed with moderate mean (Supplementary Fig. S1; Table 2) and ranged between 0.17–0.88 in men and 0.19–0.96 in women.

**Table 2:** Descriptive statistics of the Healthy Food Diversity (HFD)-Index and energy-adjusted intake of macro- and micronutrients, dietary fibre and cholesterol across the tertiles of the HFD-Index among 10,551 men and 16,080 women from the EPIC-Potsdam study population at baseline.

Characteristic	Total	Men			Women		
		Tertile 1	Tertile 2	Tertile 3	Tertile 1	Tertile 2	Tertile 3
Number	26,591	3502	3504	3505	5359	5361	5360
HFD-Index and its components							
HFD-Index [mean (SD)]	0.49 (0.10)	0.35 (0.05)	0.46 (0.02)	0.56 (0.05)	0.41 (0.06)	0.52 (0.02)	0.61 (0.04)
Berry-Index	0.95 (0.93, 0.96)	0.94 (0.91, 0.95)	0.95 (0.94, 0.96)	0.95 (0.93, 0.96)	0.95 (0.92, 0.96)	0.96 (0.94, 0.96)	0.95 (0.93, 0.96)
Health Value [mean (SD)]	0.52 (0.10)	0.38 (0.05)	0.48 (0.03)	0.60 (0.06)	0.44 (0.05)	0.55 (0.03)	0.65 (0.06)
Energy intake (kcal/day)							
Total energy [mean (SD)]	2115 (666)	2608 (747)	2406 (636)	2272 (630)	2039 (627)	1889 (522)	1800 (508)
Nutrient intake (per 1000 kcal)							
Total protein (g/d)	36 (32, 40)	36 (32, 39)	36 (33, 40)	35 (32, 39)	36 (33, 40)	36 (33, 40)	35 (32, 39)
Total fat (g/d)	39 (35, 43)	40 (36, 45)	39 (35, 43)	37 (33, 41)	41 (37, 45)	40 (36, 43)	37 (33, 41)
Saturated fatty acids (g/d)	16 (14, 18)	17 (14, 19)	16 (14, 18)	14 (12, 16)	17 (15, 19)	16 (14, 18)	15 (13, 16)
MUFA (g/d)	13 (12, 15)	14 (12, 16)	14 (12, 15)	13 (11, 14)	14 (13, 15)	13 (12, 15)	13 (11, 14)
PUFA (g/d)	7 (6, 8)	6 (5, 8)	7 (6, 8)	7 (6, 8)	7 (5, 8)	7 (6, 8)	7 (6, 8)
Cholesterol (mg/d)	142 (121, 165)	149 (127, 172)	144 (124, 164)	132 (113, 153)	151 (131, 174)	145 (125, 167)	132 (111, 153)
Total carbohydrates (g/d)	112 (101, 122)	105 (95, 116)	106 (96, 116)	112 (101, 122)	112 (102, 121)	113 (104, 122)	119 (109, 129)
Disaccharides (g/d)	33 (25, 42)	29 (22, 38)	28 (22, 36)	30 (23, 38)	37 (28, 46)	35 (28, 42)	36 (29, 44)
Dietary fibre (g/d)	11 (9, 12)	9 (8, 10)	10 (9, 11)	11 (9, 12)	10 (9, 11)	11 (9, 13)	12 (11, 15)
Calcium (mg/d)	368 (297, 454)	310 (245, 404)	315 (259, 392)	323 (270, 389)	406 (325, 518)	398 (331, 476)	396 (334, 469)
Magnesium (mg/d)	159 (145, 175)	146 (134, 160)	154 (143, 167)	163 (151, 177)	152 (139, 168)	160 (147, 175)	171 (157, 188)
Vitamin A (mg/d)	0.7 (0.6, 0.9)	0.6 (0.5, 0.8)	0.7 (0.5, 0.8)	0.7 (0.5, 0.8)	0.7 (0.6, 0.8)	0.7 (0.6, 0.9)	0.7 (0.6, 0.9)
Total folate (mg/d)	0.1 (0.1, 0.1)	0.1 (0.1, 0.1)	0.1 (0.1, 0.1)	0.1 (0.1, 0.1)	0.1 (0.1, 0.1)	0.1 (0.1, 0.1)	0.1 (0.1, 0.1)
Vitamin C (mg/d)	56 (42, 75)	33 (27, 40)	45 (38, 53)	64 (53, 79)	47 (38, 56)	63 (54, 74)	88 (74, 107)
Vitamin E (mg/d)	5.5 (4.8, 6.3)	4.6 (4.0, 5.4)	5.1 (4.5, 5.8)	5.5 (4.9, 6.3)	5.3 (4.6, 6.0)	5.8 (5.1, 6.5)	6.2 (5.5, 7.1)
Supplementation (yes, %)							
Mineral supplementation	11.9	6.9	9.0	9.9	12.8	13.4	15.9
Vitamin supplementation	17.1	13.3	14.9	16.8	17.8	17.4	20.0

Data are shown as median (interquartile range) unless otherwise stated.

While the Berry-Index did not differ between the groups of the HFD-Index, the Health Value of the diet was higher in those participants with higher HFD-Index compared to those with lower HFD-Index (Table 2). Both sexes with higher HFD-Index had lower intakes of total energy and lower energy-adjusted intakes of total fat, saturated fatty acids and cholesterol compared to those with lower HFD-Index. The energy-adjusted intake of total protein, total carbohydrates and disaccharides did not differ across the tertiles of the HFD-Index, but women had slightly higher intakes of total carbohydrates and disaccharides compared to men. With regard to micronutrients, men and women with higher compared to lower HFD-Index had higher intakes of Vitamin C, Vitamin E and magnesium, and were more likely to take mineral and vitamin supplements. Overall, women had higher intakes of vitamins and minerals than men (Table 2). The energy-adjusted intake of food groups across the whole study population as well as sex-stratified across the tertiles of the HFD-Index is presented in Supplementary Table S1. Both men and women with higher HFD-Index compared to those with lower HFD-Index were more likely to eat vegetables and fruits, wholemeal products and low-fat animal-foods, but were less likely to eat snacks and sweets, and animal foods with normal or high fat content (Supplementary Table S1).

The HFD-Index was strongly positively correlated with the Health Value (Spearman correlation coefficient r_S_ = 0.99), but a very weak correlation was found between the HFD-Index and the Berry-Index (r_S_ = 0.19; Supplementary Table S2). The correlation results between the HFD-Index, Health Value, Berry-Index, nutrient intake, DASH and MedPyr are presented in Supplementary Table S2.

### Healthy food diversity and incidence of type 2 diabetes, myocardial infarction and stroke

During a mean follow-up time of 10.5 years, 1537 participants developed T2D, whereas during a mean follow-up time of 10.7 years, 376 participants developed MI and 412 developed stroke.

The proportional hazards assumption was fulfilled for all outcomes of interest according to Schoenfeld residuals calculated separately for each outcome in adjustment model 3 (Supplementary Figs. S2 to S4). The assumption of a linear association between the HFD-Index and the three outcomes of interest was not fulfilled when investigating incident MI in women (p = 0.0171; Supplementary Figs. S5 to S7). Therefore, the results for stroke in women were only presented across the tertiles of the HFD-Index.

Table 3 shows the longitudinal associations between the HFD-Index and the incidence of T2D, MI and stroke. There was no association between adherence to the HFD-Index and the incidence of T2D in men. In women, an inverse but statistically non-significant association was observed between the HFD-Index and the incidence of T2D after adjusting for age [HR highest versus lowest HFD tertile (95% CI): 0.89 (0.74, 1.07); HR per 1 SD: 0.92 (0.85, 1.00)]. After further adjustments for socioeconomic, lifestyle and medical covariates in model 2 and 3, the association was attenuated [HR highest versus lowest HFD tertile (95% CI): 0.92 (0.77, 1.11); HR per 1 SD: 0.94 (0.87, 1.02) for model 2, and HR highest versus lowest HFD tertile: 0.97 (0.80, 1.17); HR per 1 SD: 0.97 (0.89, 1.05) for model 3] (Table 3).

**Table 3:** Longitudinal association between adherence to the Healthy Food Diversity (HFD)-Index and the incidence of type 2 diabetes, myocardial infarction and stroke among men and women from the EPIC-Potsdam study population.

	Hazard ratio (95% confidence interval)			
	Tertile 1	Tertile 2	Tertile 3	Per 1 SD
Type 2 diabetes				
Men (N = 9695)				
Cases, n/person-years	287/33,512	259/32,990	307/32,179	853/98,681
Model 1	Ref.	0.86 (0.72, 1.01)	1.02 (0.87, 1.20)	1.01 (0.94, 1.09)
Model 2	Ref.	0.90 (0.76, 1.07)	1.08 (0.92, 1.28)	1.04 (0.97, 1.12)
Model 3	Ref.	0.90 (0.76, 1.07)	1.02 (0.87, 1.21)	1.02 (0.95, 1.10)
Women (N = 15,368)				
Cases, n/person-years	233/54,760	230/54,497	221/54,415	684/163,672
Model 1	Ref.	0.93 (0.77, 1.11)	0.89 (0.74, 1.07)	0.92 (0.85, 1.00)
Model 2	Ref.	0.96 (0.80, 1.15)	0.92 (0.77, 1.11)	0.94 (0.87, 1.02)
Model 3	Ref.	0.97 (0.80, 1.16)	0.97 (0.80, 1.17)	0.97 (0.89, 1.05)
Myocardial infarction				
Men (N = 10,037)				
Cases, n/person-years	87/35,531	87/35,720	87/34,754	261/106,005
Model 1	Ref.	0.91 (0.67, 1.22)	0.92 (0.68, 1.24)	0.96 (0.84, 1.09)
Model 2	Ref.	1.00 (0.74, 1.36)	1.03 (0.76, 1.40)	1.02 (0.90, 1.17)
Model 3	Ref.	0.99 (0.73, 1.34)	0.98 (0.72, 1.33)	1.00 (0.88, 1.14)
Women (N = 15,974)				
Cases, n/person-years	36/57,466	41/57,862	38/57,492	115/172,820
Model 1	Ref.	1.04 (0.66, 1.63)	0.97 (0.61, 1.53)	Non-linearity
Model 2	Ref.	1.20 (0.76, 1.89)	1.13 (0.71, 1.80)	
Model 3	Ref.	1.18 (0.75, 1.86)	1.10 (0.69, 1.75)	
Stroke				
Men (N = 10,335)				
Cases, n/person-years	86/36,197	95/36,454	56/36,203	237/108,854
Model 1	Ref.	0.96 (0.72, 1.29)	0.56 (0.40, 0.79)	0.83 (0.72, 0.95)
Model 2	Ref.	0.98 (0.73, 1.32)	0.56 (0.40, 0.79)	0.83 (0.72, 0.95)
Model 3	Ref.	0.94 (0.70, 1.27)	0.52 (0.37, 0.74)	0.80 (0.70, 0.92)
Women (N = 15,930)				
Cases, n/person-years	44/57,326	64/57,503	67/57,229	175/172,058
Model 1	Ref.	1.34 (0.91, 1.97)	1.42 (0.97, 2.08)	1.18 (1.00, 1.39)
Model 2	Ref.	1.39 (0.94, 2.05)	1.47 (1.00, 2.16)	1.20 (1.01, 1.41)
Model 3	Ref.	1.37 (0.93, 2.02)	1.44 (0.98, 2.12)	1.20 (1.01, 1.42)

Hazard Ratios (HR) and 95% confidence intervals (CI) were calculated by Cox proportional hazards regression across the tertiles of the HFD-Index and per 1 standard deviation (SD) of the HFD-Index. Ref. = reference group.

Model 1: adjusted for age; Model 2: Model 1 + education, occupation, smoking status, alcohol intake, total energy intake, physical activity; Model 3: Model 2 + prevalent hypertension, body mass index, residuals of waist circumference regressed on body mass index, and vitamin supplementation (only type 2 diabetes) or prevalent type 2 diabetes and hyperlipidaemia (only myocardial infarction and stroke).

There was no association between the HFD-Index and the incidence of MI in both sexes (Table 3). However, men with higher adherence to the HFD-Index had a lower incidence of stroke in adjustment model 3 [HR highest versus lowest HFD tertile (95% CI): 0.52 (0.37, 0.74)]; per 1 SD increase in HFD-Index: 0.80 (0.70, 0.92)]. In contrast, women with higher adherence to the HFD-Index were at higher risk of stroke, which was robust across all adjustment models [model 3: HR highest versus lowest HFD tertile (95% CI): 1.44 (0.98, 2.12); HR per 1 SD: 1.20 (1.01, 1.42)].

#### Analysis of effect measure modification

The results for the analysis of effect measure modification by sex are presented in Supplementary Table S3. The HFD-Index was sex-specifically dichotomised based on the median HFD-Index of 0.46 for men and 0.52 for women. Based on the relative excess risk due to interaction (RERI), effect measure modification was absent in the association of the HFD-Index with T2D and MI in the multiple-adjusted model 3 [RERI (95% CI): 0.11 (− 0.17, 0.39) and 0.03 (− 0.63, 0.69), respectively]. Also, no statistically significant interaction with sex was found for the association between the HFD-Index and either T2D or MI when an interaction term was investigated (p = 0.4923 and p = 0.6796, respectively). For stroke, we found a significant effect measure modification of the HFD-Index across strata of sex on the additive scale [RERI (95% CI): − 1.18 (− 1.93, − 0.43)] and after including a multiplicative interaction term in the multiple-adjusted model (p = 0.0007). With regard to stroke risk in women, the results for the analysis of effect measure modification by age are presented in Supplementary Table S4. An interaction by age was absent on additive [RERI (95% CI): − 0.08 (− 0.81, 0.64)] and multiplicative scale (p = 0.5158).

#### Sensitivity analyses

After the exclusion of incident cases occurring during the first two years of follow-up, the results remained largely consistent or even became stronger (Supplementary Table S5). For example, in men, a higher HFD-Index was associated with a decreased risk of MI compared to a lower HFD-Index, although not statistically significant [HR highest versus lowest HFD tertile (95% CI): 0.79 (0.56, 1.12)]. For stroke, the positive association was more pronounced in women after these exclusions.

The results of the Cox regression analysis investigating the separate associations of the two components of the HFD-Index—Berry-Index and Health Value—and the three outcomes of interest are presented in Supplementary Table S6. In men, a 1 SD difference in Berry-Index was related to a lower risk of developing T2D and MI after adjustment in model 3 [HR per 1 SD (95% CI): 0.94 (0.88, 0.99) and 0.93 (0.85, 1.03), respectively]. The associations between the Berry-Index and stroke were markedly different in both sexes when being compared to the associations between the HFD-Index and stroke: The Berry-Index was largely unrelated to stroke. In contrast, relatively similar associations were observed for the Health Value compared to the HFD-Index with all outcomes of interest (Supplementary Table S6).

The results of the age-stratified analysis on stroke in women are shown in Supplementary Table S7. In women being equal to or above 51 years of age, a higher HFD-Index was associated with a higher incidence of stroke [HR per 1 SD (95% CI): 1.24 (1.01, 1.51)]. In women of younger age (< 51 years of age), associations between the HFD-Index and risk of stroke were weaker and non-significant.

## Discussion

Eating a healthful and diverse diet may be beneficial for health, but clear supporting evidence is lacking^11–13^. Previous studies rarely combined diet diversity and diet quality, which underlines the need to study a multidimensional measure of healthy food diversity in relation to health outcomes^11^. Therefore, we investigated longitudinal associations between the adherence to the HFD-Index and the incidence of T2D, MI and stroke in middle-aged German men and women during a mean follow-up of 10.6 years. We identified a higher healthy food diversity to be inversely associated with the incidence of stroke in men, which was robust across different adjustment models. In women, a harmful association was observed between the adherence to the HFD-Index and incident stroke. No clear evidence was found for an association between healthy food diversity and the incidence of either T2D or MI.

So far, only one study examined the association between the HFD-Index and major chronic diseases^21^. A prediction study investigating associations of diet diversity with 10-year atherosclerotic cardiovascular risk in US adults revealed no association between the HFD-Index and cardiovascular risk^21^. In a cross-sectional study, vegetable variety calculated based on the HFD-Index approach was not clearly associated with prevalent chronic diseases among 39,000 US adults, and vegetable variety was only inversely associated with prevalent coronary heart disease in US men, but not in women^22^. Comparison of our results with the two above-mentioned studies is highly limited, because the previous studies did not longitudinally investigate an aetiological objective or only applied vegetable variety without reflecting the total healthy food diversity^21,22^.

Most previous prospective studies on diet diversity or diet quality in relation to chronic disease endpoints have investigated these two concepts separately^11,13,14^. Two recent literature reviews found overall inconclusive findings for the relation of diet diversity with T2D and CVD^11,13^. Instead, diet quality assessed by different diet quality indices, was associated with lower risks of CVD and T2D in a recent meta-analysis including more than 3.2 million participants^14^. Pooled risk ratios (95% CI) for highest diet quality were 0.81 (0.78, 0.85) for T2D risk and 0.80 (0.78, 0.82) for risk of CVD incidence or mortality compared to lowest diet quality, which was robust across sub-group analyses stratifying by type of diet quality index^14^. National dietary guidelines define diet quality as a high intake of potentially health-beneficial foods and nutrients and a low intake of those being potentially health-detrimental^4–7^. Existing diet diversity indicators usually count the number of either all consumed food items or only selected food items^11^. Food group-based diet diversity indicators sum up the number of different food groups consumed during a specified reference period, but there is no definition on the allocation of food items into food groups or the amount of food groups^11^. These diet diversity measures do not identify whether diversity is driven by healthy or unhealthy foods, nor do they consider the proportional distribution of foods and food groups in the diet^8,15,16^. Thus, existing diet diversity indicators hardly reflect overall diet quality, which limits the interpretation of associations between pragmatically defined diet diversity and health outcomes^11,17^.

Plausible biological mechanisms driving a potential beneficial impact of healthy food diversity on cardiometabolic health have been previously proposed^13,19^. For example, greater diet diversity within healthier - low-energy and nutrient-dense - foods may increase their intake and simultaneously reduce the intake of less healthy alternatives (energy-dense and nutrient-poor)^19,23^. Studies focusing on fruit and vegetable variety and cardiovascular health found a strong relationship between fruit and vegetable variety and their amount of intake^22,24,25^. Higher healthy food diversity could potentially promote the intake of bioactive compounds comprising dietary fibre, phytochemicals and antioxidants^18,22^. These have been associated with a lower incidence of cardiometabolic disease incidence via multiple mechanisms such as regulation of blood pressure, lipoprotein profiles and blood glucose, and reduction of insulin resistance, inflammation and oxidative stress^18,22^. A higher intake of plant-based foods being lower in energy and glycaemic index but higher in dietary fibres may promote physiological satiety, which in turn could facilitate adherence to a low-energy, nutrient-dense diet^18,19,26^. A large variability in healthy foods may increase attractiveness and decrease restrictiveness of reduced-energy and healthy dietary patterns, which could enhance dietary satisfaction^19^ and facilitate adherence to a health-beneficial dietary behaviour^19^.

In light of the possible beneficial influence of healthy food diversity and the lack of knowledge regarding the relation of healthy food diversity to chronic disease risk, we extended the literature by investigating the performance of the multidimensional HFD-Index with regard to chronic diseases. The HFD-Index simultaneously considers the diversity and healthiness of the diet including quality and proportionality of the total food intake according to the dietary guidelines of the German Nutrition Society^15^. Compared to traditional diet diversity indicators, the HFD-Index has the advantage to increase not only with increasing number of food items, but also if the relative contribution of healthier food groups to total food consumption increases^15^. Indeed, we showed that both men and women with higher HFD-Index compared to those with lower HFD-Index had higher intakes of healthy foods such as vegetables, fruits and wholemeal products and lower intakes of unhealthier foods like sweets and snacks. The HFD-Index was previously validated using energy-adjusted correlations with diet quality indicators and overall higher correlated with both nutrient supply (e.g. folate, vitamin E and C, and dietary fibre) and serum HDL cholesterol than traditional diversity measures (Berry-Index and Count-Index)^15^. In our study, the HFD-Index was overall stronger correlated with nutrient intake than the Berry-Index. However, we did not find any evidence for beneficial associations between the HFD-Index and T2D and MI. This was potentially driven by limitations of the HFD-Index. First, the Berry-Index has the limitation, that it increases with equal distribution of food items regardless of the diet`s healthiness, which is only reduced but not eliminated by incorporation of the Health Value^15^. The Berry-Index did not differ between men and women across the tertiles of the HFD-Index and only showed small variation across the total population, so that the HFD-Index hardly reflected diet diversity in our study population. Second, the majority of food groups were highly aggregated in the Health Value component and lacked separation from other food groups. For example, processed meat was either assigned to the food subgroup “Fish/low-fat meat/low-fat meat products”, “Meat products/sausages/eggs” or “Bacon”. Thus, processed meat items were mixed up with fish, non-processed meat and eggs, but recent evidence clearly demonstrated harmful associations between the intake of processed meat and chronic disease risk^27–29^. The potential detrimental effects of processed meat may be masked when it is not separated from other food groups, resulting in the effects of the food groups on disease risk cancelling each other out. Other examples, which illustrate the broad grouping of food items in the HFD-Index are: no distinction between (a) fermented and non-fermented dairy products, (b) red and white meat, and (c) fish and fatty marine fish^30^. In contrast to the two main food groups “plant foods” and “animal foods”, the “fats and oils” food group was further divided into more specific categories.

The Health Value of the HFD-Index was defined in concordance with the German dietary guidelines^5,15^. A previous study investigating the adherence to the German dietary guidelines reflected by the German Food Pyramid Index (GFPI) and chronic disease risk within the EPIC-Potsdam study population found an inverse association between the GFPI and CVD risk in men and a trend towards a harmful association with CVD risk in women^31^. Similar to our study, clear associations between the GFPI and T2D risk were not found^31^. The comparable results are potentially driven by similar dietary guidelines being used to define diet quality in both studies. The dietary guidelines at the time of development of the HFD-Index are unlikely based on the most current evidence on food group associations with chronic disease risk^30^. As a result, the potential benefit of an increased intake of healthy foods (e.g. wholegrain bread and cereals, fermented dairy products, fruits, vegetables, fatty fish, vegetable oils) might have been outweighed by the unhealthy foods (e.g. red meat, processed meat, sugar-sweetened beverages)^13,30^. We investigated the correlation between the HFD-Index and two diet quality scores, DASH and MedPyr, and identified only weak to moderate correlations. This is potentially due to methodological differences not only in the underlying dietary recommendations but also in the amounts and separations of food groups. The Health Value incorporated in indices reflecting both diversity and diet quality, like the HFD-Index, could be defined in different ways. Updated food-based dietary guidelines would allow integrating more recent dietary guidance. Alternatively, other measures of diet quality, which have been more clearly related to reduced risk of chronic diseases could be used, e.g. as those systematically summarised by Morze et al*.*^14^.

In our study, the Health Value mainly drove the overall associations of the HFD-Index, while the Berry-Index contributed little. Investigating both components individually highlighted that the Berry-Index was inversely associated with the incidence of T2D in men, while we neither found any association with T2D in women nor with MI or stroke in both sexes. Similar to our results, previous studies already reported an inverse or null association between diet diversity assessed by different diet diversity scores and type 2 diabetes^9,10,32–34^. To our knowledge, only one longitudinal study investigated the association between diet diversity and cardiovascular diseases, and identified mixed results^35^. Since previous studies used different methodologies to assess diet diversity, study results are hardly comparable^9,10,13,32–35^. We need a unique definition on how to assess and conceptualise diet diversity, which could further be implemented in the HFD-Index to improve its ability to reflect diet diversity.

Another strength of this work lies within the approach of adjustment for potential confounders. The decision on which confounders and covariates need to be considered in the analysis was based on outcome-specific directed acyclic graphs (DAGs), which were drawn by using previous literature^36–41^. However, the relationships between the exposure, covariates and outcomes are highly complex and some path directions are not entirely clear. On the one hand, exposure and covariates have been assessed at the same time point making assumptions of arrows in the DAG unavoidable because temporal sequence of exposure and many covariates could not be established. For example, the participant`s healthy food diversity could have potentially caused prevalent hypertension, but it could also be true vice versa assuming that participants with prevalent hypertension could have changed their dietary behaviour due to recommendations made by their physician. Thus, prevalent hypertension could be a confounder of the causal path from HFD-Index to incident T2D, resulting in confounding bias when not being adjusted for in the analysis^42^. It could also be a mediator introducing overadjustment bias when being adjusted for in the analysis^42^. Prevalent hypertension might not even cause incident T2D but is rather a comorbidity and both diseases share common risk factors^43^. With regard to incident MI and stroke, medical conditions at baseline such as abdominal obesity, prevalent hypertension, elevated blood lipid levels and prevalent T2D could have mediated or confounded the effect of HFD-Index on risk of MI or stroke based on the assumed direction of paths. Indeed, these conditions could have also introduced collider bias when being adjusted for, assuming that for example prevalent T2D is caused by HFD-Index and some other factor (e.g. obesity), and both prevalent T2D and the other factor cause incident MI^42,44^. Due to the many uncertainties in causal paths and the complex pathogenesis of the three outcomes of interest, the adjustment strategy was not restricted to one adjustment model but based on three adjustment models^45^. The results were robust across the three different adjustment models, so that in our study both collider and overadjustment bias are presumably unlikely.

Due to the potential of unmeasured or unknown confounding factors, we could not rule out residual confounding entirely. For example, sex-specific differences in stroke risk have just recently received more attention and the importance of sex-specific risk factors, e.g. pregnancy complications and late menopause, have been identified^46^. As the applied adjustment strategy was the same for men and women, the considered confounding variables were potentially not sufficient to minimise confounding bias in the analysis of women. Since we adjusted for several stroke risk factors, which are also associated with menopause such as waist circumference, BMI, hyperlipidaemia, prevalent hypertension and prevalent T2D^47^, we speculate that confounding bias introduced by the lack of sex-specific adjustment sets to be unlikely an explanation for the contrasting associations between men and women. Future research should investigate the importance of sex-specific adjustment strategies on diet-related risk for stroke^46^.

We stratified the women’s analysis on stroke by age with 51 years being the cut-off age and checked for potential interaction by age^47^. Although interaction by age was statistically absent, the harmful association between the HFD-Index and risk of stroke appeared more pronounced in women of older age. The potentially modifying role of menopausal status on the effect of diet on stroke risk needs further investigations.

Further strengths and limitations of the current study should be mentioned. A clear advantage was the use of data from a prospective cohort study with a mean follow-up of almost 11 years. The verification process of incident cases increased the validity of our observations. The possible influence of reverse causation was addressed by excluding prevalent and non-verified, outcome-specific cases in the main analyses as well as incident cases within the first 2 years of follow-up in sensitivity analyses^48^. Our results indicate that reverse causation is an unlikely explanation for the observed associations. Although EPIC-Potsdam reflects a general population sample, our results may have limited generalisability because the study participants were mostly well-educated and of similar cultural Northern German background. Also, dietary exposure and covariates were self-reported and thus prone to misreporting^49^. We partly addressed this limitation by excluding those participants with implausible energy intakes. Moreover, the FFQ presents a finite list of food items, which potentially underestimates the habitual diet diversity^50^. Dietary intake was only assessed once at baseline, so that we cannot rule out potential dietary changes over time, which would have biased the results. We not only recommend using a dietary assessment tool of greater ability to assess diet diversity such as multiple 24-h dietary recalls but also measuring dietary intake at several time-points during follow-up.

In conclusion, our study was the first longitudinal study, which investigated the aetiological association of the HFD-Index to measure a healthy and diverse diet with major chronic diseases. In this middle-aged population from Germany, there was no clear association between healthy food diversity and T2D or MI incidence, but we found a beneficial association between HFD-Index and stroke in men and a harmful association in women. We highlighted the importance of future studies thoroughly considering adjustment approaches especially with regard to stroke emphasising the need for sex-stratification. A deeper and systematic insight into the combination of diet diversity and diet quality in longitudinal relation to health outcomes among different populations would add valuable contribution to future investigations.

## Methods

### Study design and population

The EPIC-study, a multi-centre prospective cohort study, was initiated in 1992 to investigate the association between diet and risk of cancer, other chronic diseases and overall mortality in ten European countries^51,52^. One of the two German study centres is located in Potsdam, where a random sample of general population was recruited via addresses provided by the registration offices^53,54^. Recruitment took place between August 1994 and September 1998 resulting in 27,548 participants at baseline (participation rate 22.7%)^54^. The target population comprised men aged 40–64 years and women 35–64 years living in Potsdam or surrounding areas^54^. From 1997 onwards, participants were actively re-invited for follow-up in approximately 2-years intervals^55^. Passive follow-up included, among others, the use of death certificates^55^. Between each follow-up, more than 90% response rate was achieved. Written informed consent was obtained from each participant at recruitment, and the study followed the recommendations of the Declaration of Helsinki. Ethical approval was given by the Ethical Committee of the Federal State of Brandenburg, Germany. Further details on recruitment and follow-up procedures of the EPIC-Potsdam study can be found elsewhere^53–55^.

**Fig. 1:**
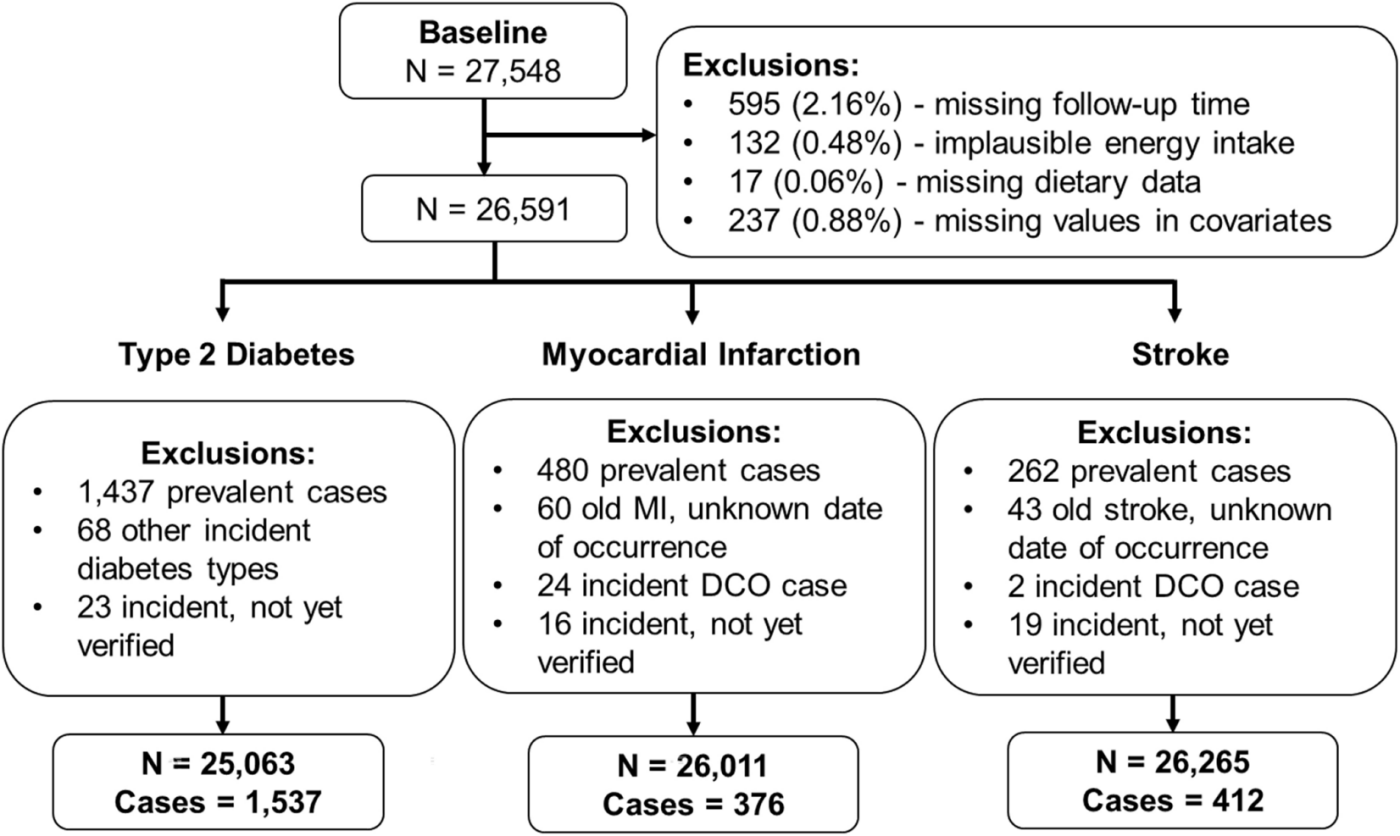
Flow diagram of the analytical EPIC-Potsdam study population.

Data until the end of follow-up 5 in 2009 was considered for the present analysis. In order to address potential sources of bias, the following exclusion criteria were applied. Participants with missing follow-up time or missing values in covariates of interest were excluded. To address potential misreporting bias of dietary intake, participants with implausibly low or high energy intake were further excluded (< 800 or > 6000 kcal/day). The analytical sample size comprised 26,591 participants prior to outcome-specific exclusions (Fig. 1). In addition, prevalent cases of T2D, MI or stroke at baseline, non-verified cases or cases with unknown date of occurrence were excluded to address potential reverse causation bias^48^. The final outcome-specific sample sizes for T2D, MI and stroke comprised 25,063, 26,011 and 26,265 participants, respectively (Fig. 1).

### Dietary assessment and exposure construction

Habitual food intake of the past 12 months was assessed at baseline using a self-administered, optically readable and semiquantitative food-frequency questionnaire (FFQ) comprising 149 single food items. The amount of each food item eaten per day was calculated using the frequency of intake and the information on portion sizes^56^. Additional questions included in the FFQ provided information on specific dietary aspects such as fat content of dairy and meat products consumed, types of fat usually used for food preparation or seasonal variation in fruit consumption^56^. Reproducibility of food group intake collected by the FFQ was estimated among 104 men and women from Western Germany, who repeatedly completed the FFQ after six months^57^. Spearman correlations of food group intake ranged between 0.49 for bread and 0.77 for meat. Further correlation coefficients were: 0.77 for fish, 0.73 for processed meat, 0.72 for salty snacks, 0.71 for sweets, 0.71 for potatoes, 0.61 for fruits, 0.61 for cheese, 0.55 for milk and milk products, and 0.54 for vegetables. In this study, the FFQ was also compared to twelve monthly 24-h dietary recalls to estimate relative validity of food group intake^57^. Deattenuated correlation coefficients varied between 0.25 for nuts and 0.74 for cakes and biscuits. For meat, the correlation was 0.67, 0.58 for cheese, and milk and milk products, 0.57 for salty snacks, 0.54 for bread, and fruits, 0.49 for potatoes, 0.42 for vegetables and 0.37 for fish^57^.

The individual`s usual food intake was used to construct the HFD-Index based on the calculation proposed by Drescher et al.^15^. More information on the construction of the HFD-Index can be found in the Supplementary Methods “Calculation of the HFD-Index”. Briefly, the HFD-Index includes the Berry-Index as a measure of dietary diversity, and a Health Value, which describes the quality and proportionality of the total food intake according to the dietary guidelines of the German Nutrition Society^5,15^. Since the Berry-Index is multiplied by the Health Value to yield the HFD-Index, a healthy food choice will be downgraded in the HFD-Index if diversity is low and vice versa^15^. The higher the HFD-Index, the larger the number of different foods consumed and the better the diet`s healthiness^15^. Every participant achieved an HFD-Index between 0 and nearly 1. For further analyses, the HFD-Index was divided into three groups approximately equal in size by using sex-stratified tertiles as borders, referred to as tertile 1, 2 and 3.

### Outcome assessment

During follow-up assessments, potential cases of a chronic disease (T2D, MI, stroke) were self-reported either via stating the respective condition, relevant medication, or an indicative reason for a reported change in dietary habits^53^. Each potential incident case was further medically verified via contacting physicians or the treating hospital to confirm the diagnosis^53,55^. Furthermore, death certificates were used to identify cases. Incident cases were coded according to the International Statistical Classification of Diseases and Related Health Problems (ICD) -10 codes: I21 for MI, I60, I61, I63 and I64 for stroke, and E11 for T2D.

### Covariate assessment

At baseline, physical measures were obtained by trained personnel in the examination centre^54^. The participant’s body mass index (BMI) was calculated using the measured weight (kg) and dividing it by the squared height (m). Waist circumference (WC; cm) and blood pressure (mmHg) were measured. Sex-specific residuals for WC regressed on BMI were calculated^58^. General information on sociodemographic, lifestyle and health condition were collected by self-administered questionnaires and computer-guided interviews^53,54^. Assessed information on sociodemographic and lifestyle included the following: age, sex, education, occupation, smoking status, alcohol intake defined as lifetime alcohol consumption pattern, total energy intake, total sports and intake of vitamin supplements. Information on prevalent hypertension was defined at baseline as systolic BP ≥ 140 mmHg, diastolic BP ≥ 90 mmHg (based on the mean of the second and third measurement^59^), antihypertensive medication use or prior diagnosis of hypertension. Hyperlipidaemia was self-reported. More details on selected covariate data collected at baseline are shown in Supplementary Table S8.

### Statistical analyses

Sociodemographic, anthropometric, lifestyle, medical and energy-adjusted dietary characteristics were investigated descriptively among the whole study population as well as across strata of sex and HFD-Index. Normally or non-normally distributed continuous variables were presented as means and standard deviations (SD) or medians and interquartile ranges (IQR), respectively. Categorical variables were depicted as relative frequencies. Spearman correlations of the HFD-Index, Berry-Index and Health Value with the intake of nutrients, dietary fibre and cholesterol were calculated to investigate the HFD-Index`s ability to reflect a healthy diet. Partial correlation was used to adjust Spearman correlation coefficients for total energy intake. Energy-adjusted Spearman correlation coefficients were calculated between the HFD-Index and two other widely used diet quality scores, the Dietary Approaches to Stop Hypertension (DASH)^60^ diet and the Mediterranean diet based on the Mediterranean Pyramid (MedPyr) recommendations^61,62^.

The longitudinal association between the HFD-Index and the risk of incident T2D, MI and stroke was separately investigated by applying survival analysis using Cox proportional hazards regression models^63^. Multivariable-adjusted hazard ratios (HRs) and 95% confidence intervals (CIs) were calculated continuously (by 1 SD of the HFD-Index) and across tertiles of the HFD-Index with the first tertile considered as the reference group. The time period between baseline age and exit age (age at diagnosis, death or censoring) was defined as the underlying time scale^63^. Potential ties in failure time were handled according to Efron approximation, which has been shown to be more precise when being compared to the Breslow approximation^64^. The proportional hazards assumption was tested sex-stratified for the multivariable-adjusted association (model 3) between the continuous HFD-Index and incident T2D, MI and stroke separately via calculating Schoenfeld residuals^63^. For all cases, a separate Schoenfeld residual is calculated for each variable in the Cox model, which is defined as the difference between the observed value of a covariate and its expected value given the risk set at the time of event^65^. Scaled Schoenfeld residuals were correlated with survival time using Pearson correlation, assuming independence between residuals and time^65^. A significant relationship between Schoenfeld residuals and survival time would discard the proportional hazards assumption for the according variable^65^. The second assumption of linear associations between the HFD-Index and outcomes of interest were checked for the multiple-adjusted model 3 by calculating restricted cubic splines^66^. Three knots were specified at the 5th, 50th and 95th percentile, and linearity was tested by the Wald χ^2^ test with three degrees of freedom.

To reduce violations of the HR due to different age-dependent risk at baseline, the models were stratified by integers of age^62^. Potential confounders and covariates that needed to be adjusted for in the regression models were identified by using previous literature^36–41^, and creating outcome-specific directed acyclic graphs (DAG)^67^ (Supplementary Figs. S8 to S10). With regard to T2D, the first model was only age-stratified, while the second model was further adjusted for education, occupation, smoking status, alcohol intake, total energy intake and physical activity, and the third model additionally considered prevalent hypertension, vitamin supplementation, BMI and residuals of WC regressed on BMI. For MI and stroke, the third adjustment model was slightly different, including prevalent hypertension, prevalent T2D, hyperlipidaemia, BMI and residuals of WC regressed on BMI. Since sex differences in the pathogenesis of T2D, MI and stroke have been discussed^46,68,69^, we assumed sex to be a modifier of the effect of HFD-Index on the outcomes of interest. Thus, all analyses were stratified for men and women and the DAGs did not include sex^70^.

Potential effect measure modification by sex and age (only for stroke in women) was further checked. First, a product term for either sex or age and continuous HFD-Index was included in the multiple-adjusted model 3 to check effect measure modification on a multiplicative scale^71^. Second, additive effect measure modification was checked by presenting effect estimates and 95% CIs for each stratum of the binary-modelled HFD-Index (low versus high HFD-Index using the sex-specific median HFD-Index as the cut-off) within either strata of sex or age using one reference category (women with high HFD-Index or women below 51 years of age, respectively)^72^. The relative excess risk due to interaction (RERI) was calculated to measure effect measure modification on the additive scale^72–74^.

Several sensitivity analyses were conducted to test the robustness of the results. First, incident cases occurring during a follow-up time of less than two years were outcome-specifically excluded in order to limit the possibility of reverse causality. With regard to the analysis of MI and stroke, early incident T2D cases with a follow-up time of less than 2 years were additionally excluded. Moreover, the continuous Cox regression were performed separately for the two components of the HFD-Index using either the Berry-Index or the Health Value as exposure. With regard to stroke, the longitudinal analysis was further stratified by age using the strata below 51 years old and equal to or above 51 years old, because women after menopause might have an increased risk of stroke^47^.

The statistical analyses were performed with SAS Version 9.4 Enterprise Guide Version 7.1 (SAS Institute Inc., NC, USA).

## Supplementary Information

Supplementary Information.

## Data Availability

The EPIC-Potsdam study data can be made available upon request directed to Prof Dr Matthias B. Schulze (mschulze@dife.de), head of the department Molecular Epidemiology at the German Institute of Human Nutrition Potsdam-Rehbruecke. Data cannot be made available in a public repository due to legal and ethical restraints.
