# Supplementary material for: Healthy food diversity and the risk of major chronic diseases in the EPIC-Potsdam study
Source: Sci Rep. 2024 Nov 19;14:28635. doi: 10.1038/s41598-024-78287-5 (PMC11577018; doi:10.1038/s41598-024-78287-5)
Supplement: Supplementary file 1 — Supplementary Information. [file 41598_2024_78287_MOESM1_ESM.docx]

# Supplementary Material

**a**


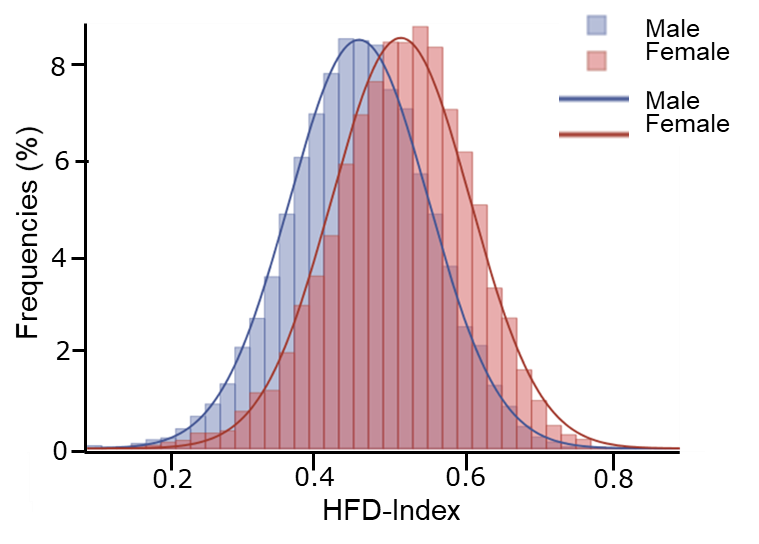


**b**


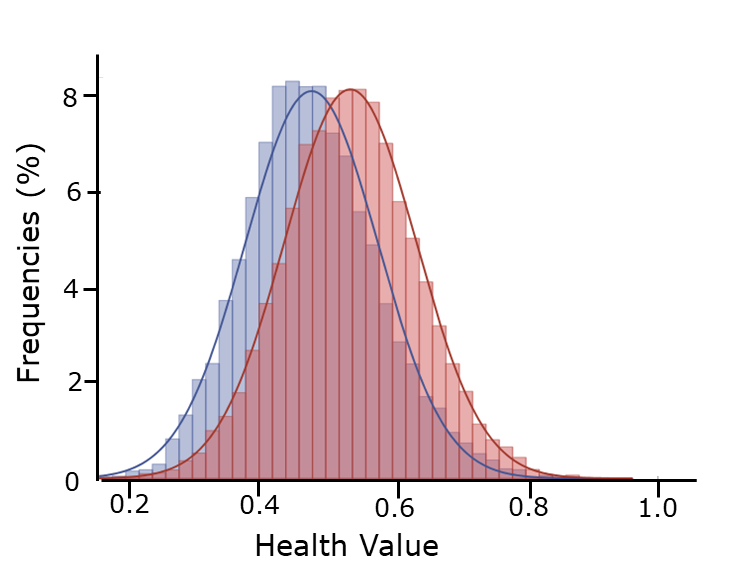

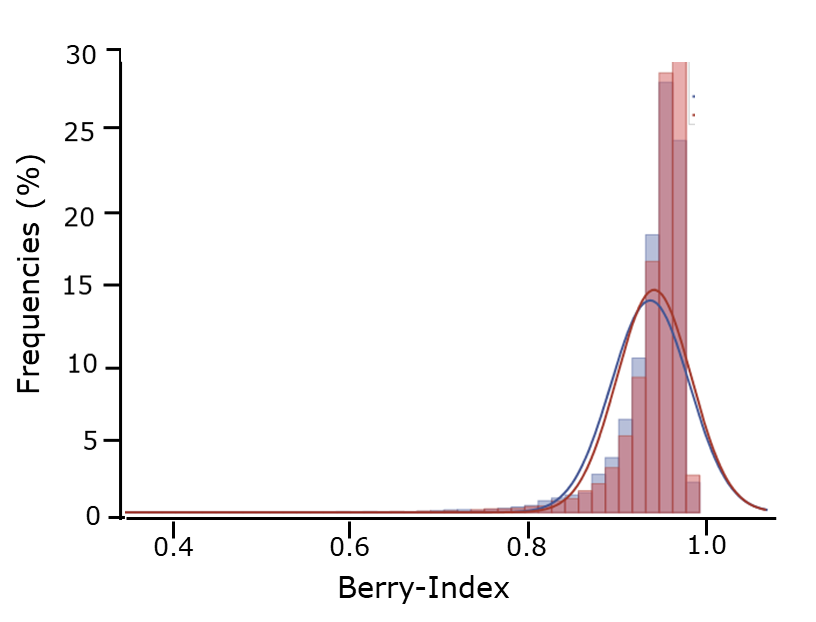


**c**

**Supplementary Figure S1.** Distribution of the (a) Healthy Food Diversity (HFD)-Index and its components (b) Health Value and (c) Berry-Index among 10,511 men and 16,080 women from the EPIC-Potsdam study population at baseline.


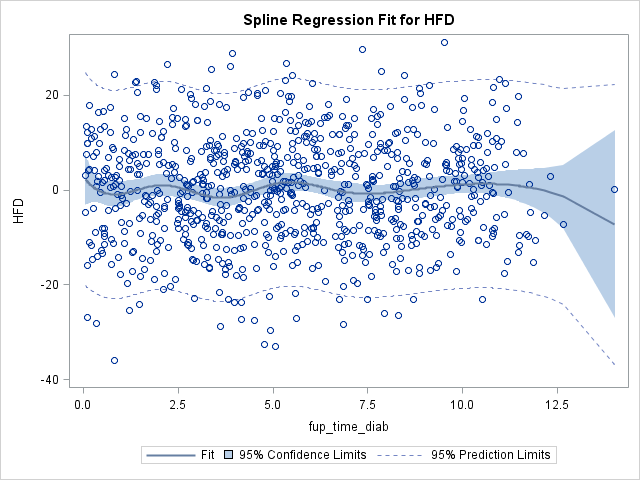


**a**


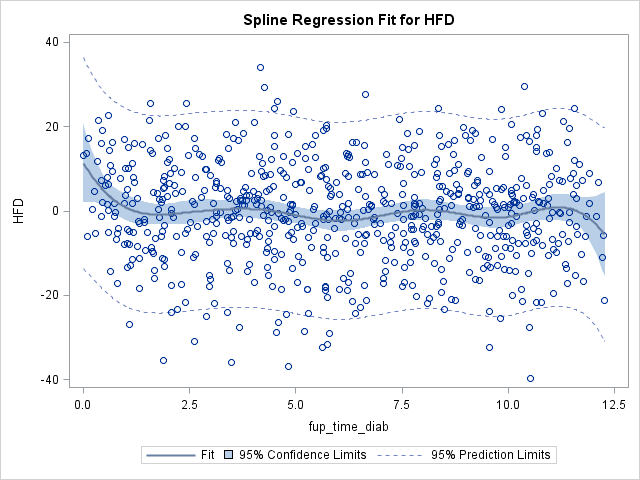


**b**

**Supplementary Figure S2.** Spline regression fit for the Schoenfeld residuals in dependency of the follow-up time (fup_time_diab) for the association between the Healthy Food Diversity (HFD)-Index and type 2 diabetes in (a) men and (b) women.

The model was adjusted for age, education, occupation, smoking status, alcohol intake, total energy intake, physical activity, prevalent hypertension, vitamin supplementation, body mass index, residuals of waist circumference regressed on body mass index (model 3). Pearson correlation coefficients between Schoenfeld residuals and follow-up time were 0.02 (p=0.5587) for men and -0.05 (p=0.1862) for women.


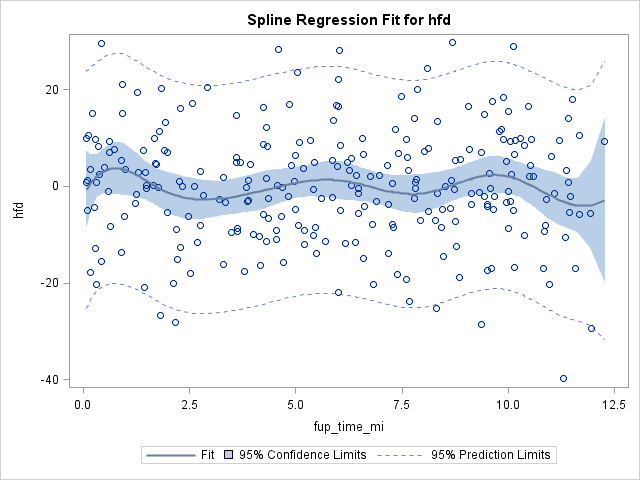


**a**


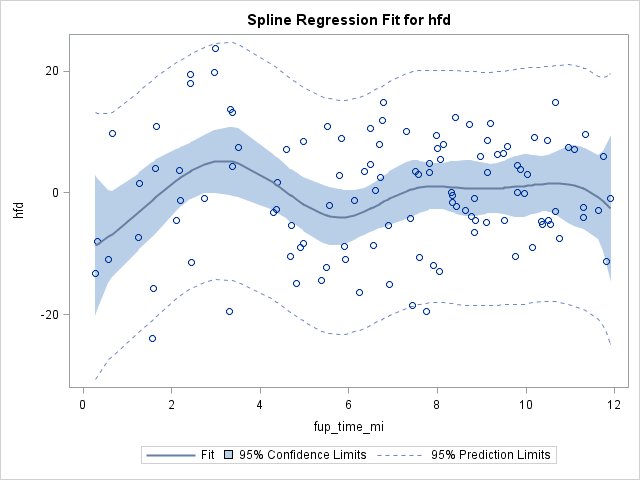


**b**

**Supplementary Figure S3.** Spline regression fit for the Schoenfeld residuals in dependency of the follow-up time (fup_time_mi) for the association between the Healthy Food Diversity (HFD)-Index and myocardial infarction in (a) men and (b) women.

The model was adjusted for age, education, occupation, smoking status, alcohol intake, total energy intake, physical activity, prevalent hypertension, prevalent type 2 diabetes, hyperlipidaemia, body mass index, residuals of waist circumference regressed on body mass index (model 3). Pearson correlation coefficients between Schoenfeld residuals and follow-up time were -0.03 (p=0.6632) for men and 0.05 (p=0.5760) for women.


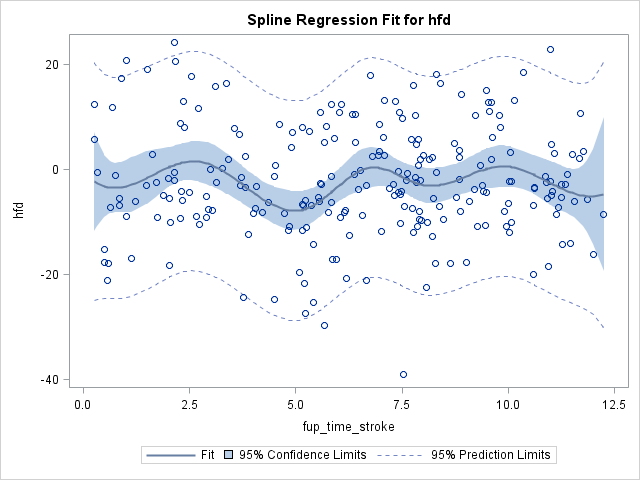


**a**


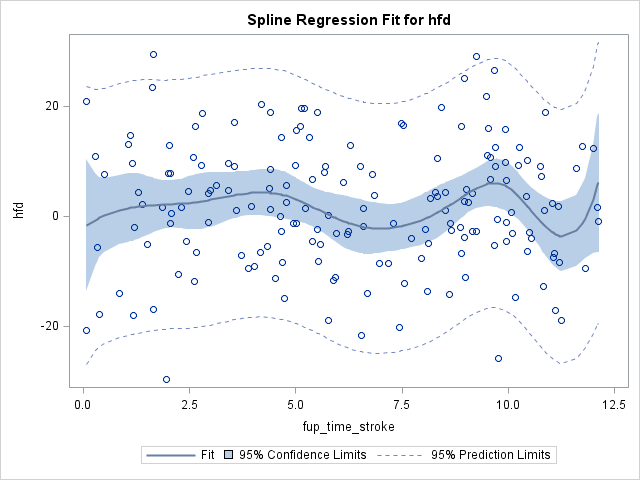


**b**

**Supplementary Figure S4.** Spline regression fit for the Schoenfeld residuals in dependency of the follow-up time (fup_time_stroke) for the association between the Healthy Food Diversity (HFD)-Index and stroke in (a) men and (b) women.

The model was adjusted for age, education, occupation, smoking status, alcohol intake, total energy intake, physical activity, prevalent hypertension, prevalent type 2 diabetes, hyperlipidaemia, body mass index, residuals of waist circumference regressed on body mass index (model 3). Pearson correlation coefficients between Schoenfeld residuals and follow-up time were -0.0001 (p=0.9988) for men and -0.005 (p=0.9430) for women.


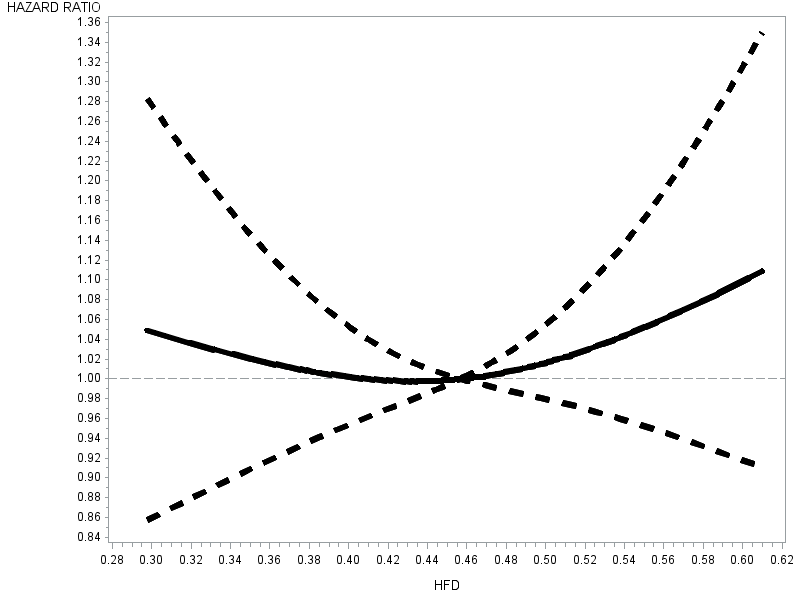

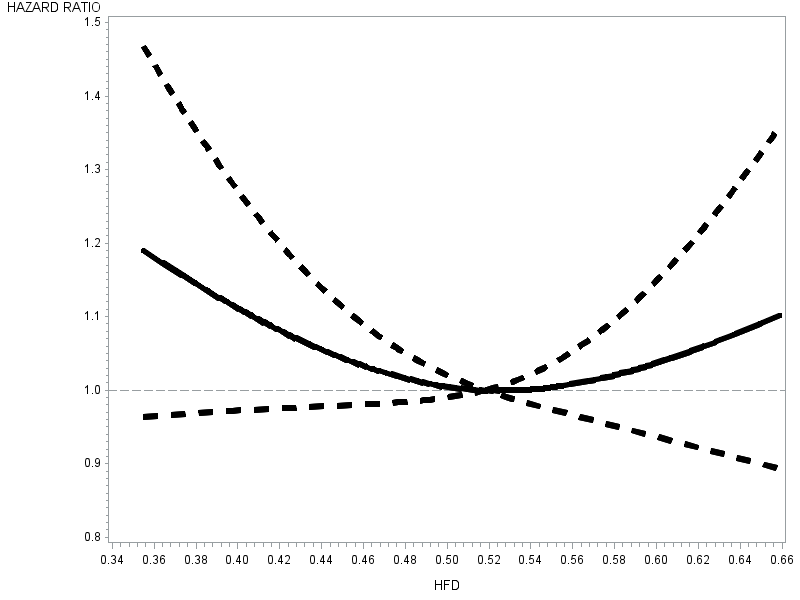


**b**

**a**

**Supplementary Figure S5.** Restricted cubic splines for the association between the Healthy Food Diversity (HFD)-Index and type 2 diabetes in (a) men and (b) women.

The model was adjusted for age, education, occupation, smoking status, alcohol intake, total energy intake, physical activity, prevalent hypertension, vitamin supplementation, body mass index, residuals of waist circumference regressed on body mass index (model 3). The p-values for linearity were 0.3593 in men and 0.1237 in women.


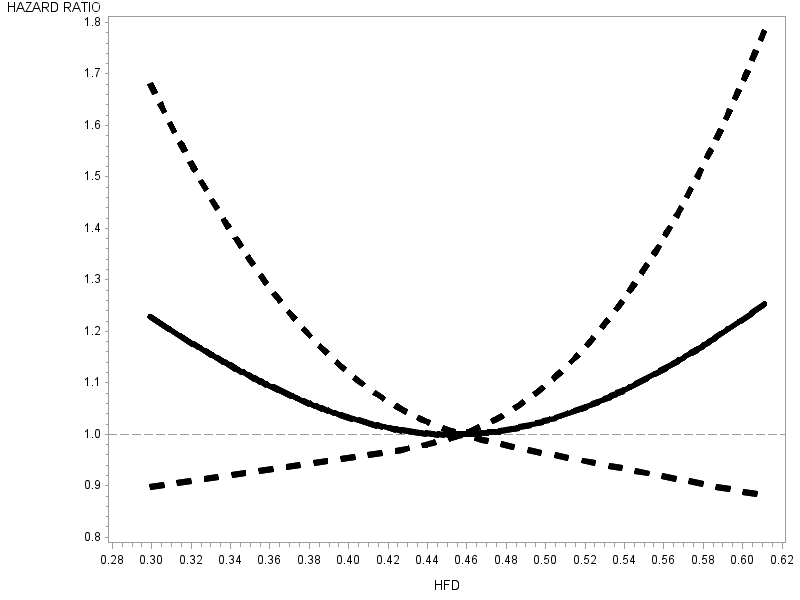

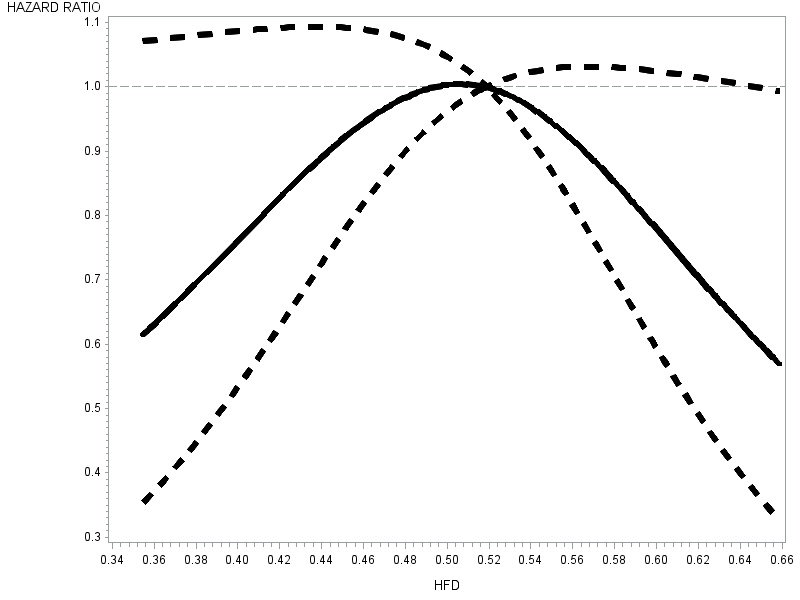


**b**

**a**

**Supplementary Figure S6.** Restricted cubic splines for the association between the Healthy Food Diversity (HFD)-Index and myocardial infarction in (a) men and (b) women.

The model was adjusted for age, education, occupation, smoking status, alcohol intake, total energy intake, physical activity, prevalent hypertension, prevalent type 2 diabetes, hyperlipidaemia, body mass index, residuals of waist circumference regressed on body mass index (model 3). The p-values for linearity were 0.1193 in men and 0.0171 in women.


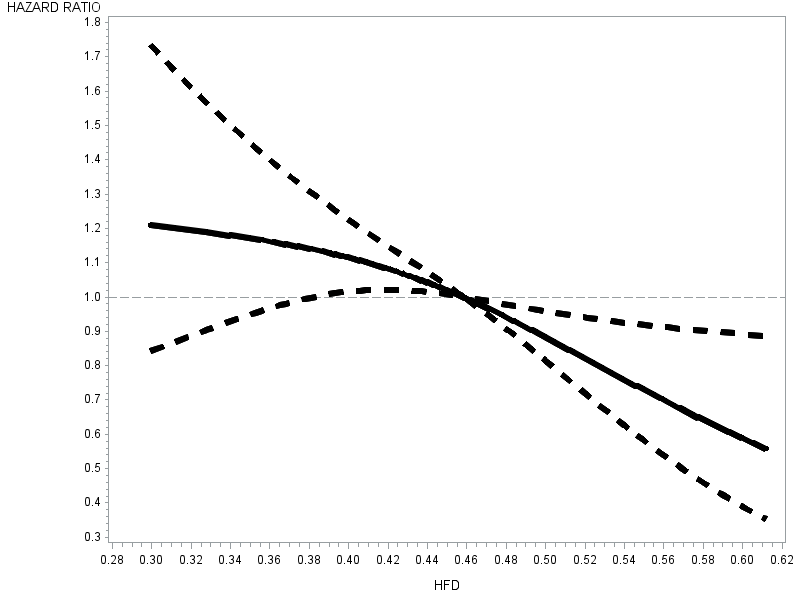

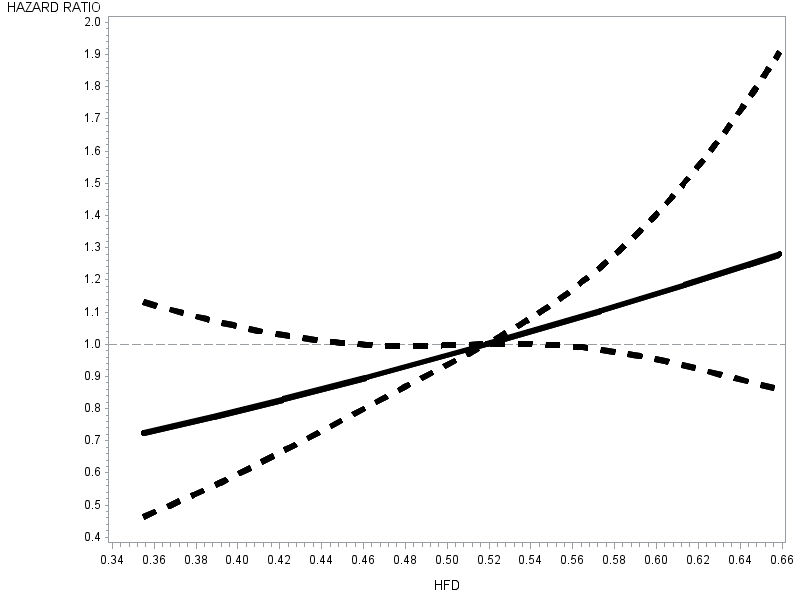


**b**

**a**

**Supplementary Figure S7.** Restricted cubic splines for the association between the Healthy Food Diversity (HFD)-Index and stroke in (a) men and (b) women.

The model was adjusted for age, education, occupation, smoking status, alcohol intake, total energy intake, physical activity, prevalent hypertension, prevalent type 2 diabetes, hyperlipidaemia, body mass index, residuals of waist circumference regressed on body mass index (model 3). The p-values for linearity were 0.2388 in men and 0.9254 in women.


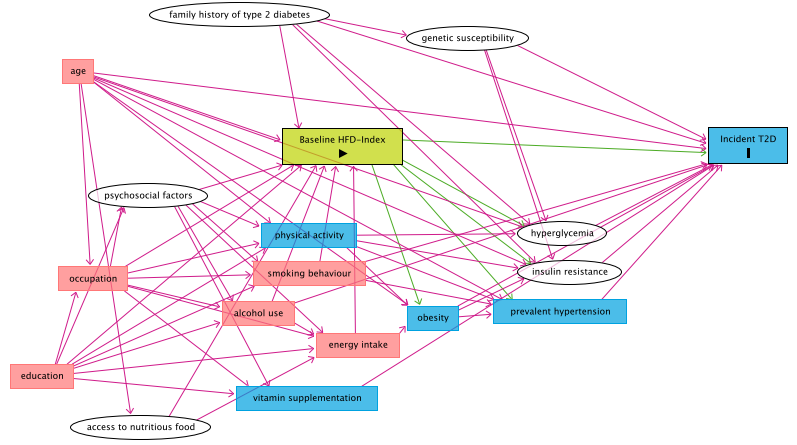


**Supplementary Figure S8.** Directed acyclic graph for the potential causal relationship between baseline Healthy Food Diversity (HFD)-Index and incident type 2 diabetes (T2D).

Legend:

| 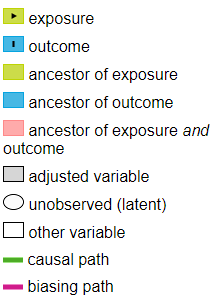 | exposure | 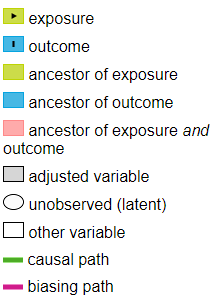 | ancestor of outcome | 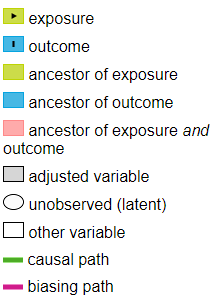 | unobserved | 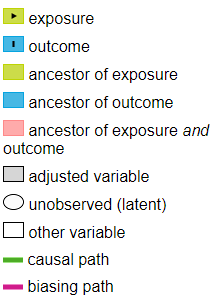 | causal path |
| --- | --- | --- | --- | --- | --- | --- | --- |
| 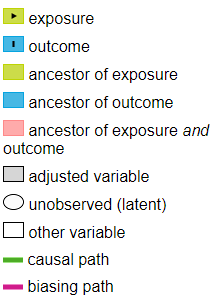 | outcome | 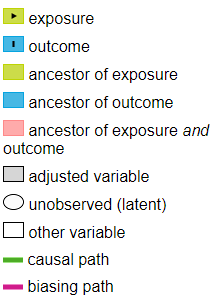 | ancestor of exposure and outcome |  |  | 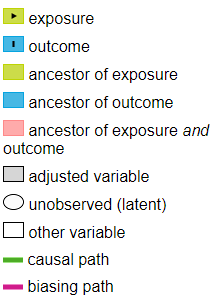 | biasing path |


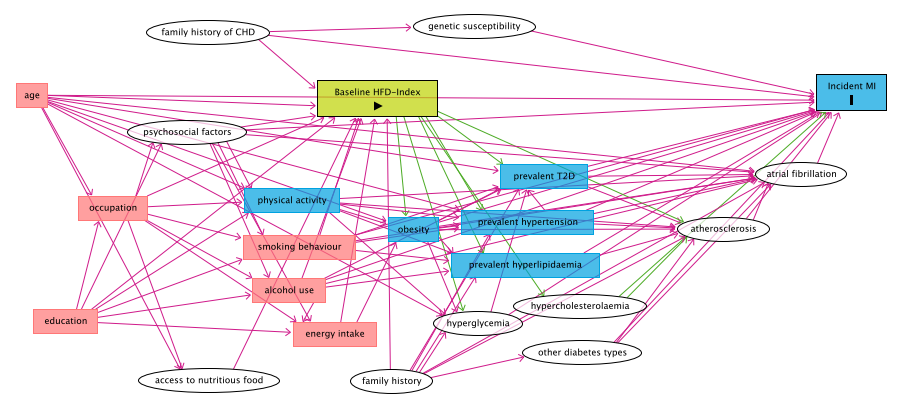


**Supplementary Figure S9.** Directed acyclic graph for the potential causal relationship between baseline Healthy Food Diversity (HFD)-Index and incident myocardial infarction (MI).

T2D = type 2 diabetes, CHD = coronary heart disease.

Legend:

| 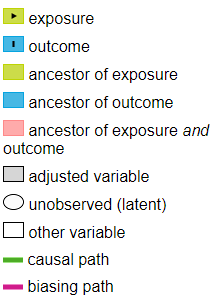 | exposure | 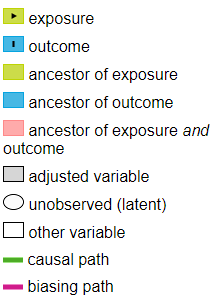 | ancestor of outcome | 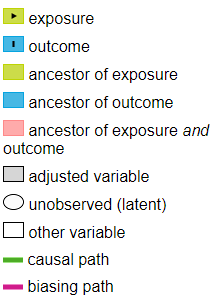 | unobserved | 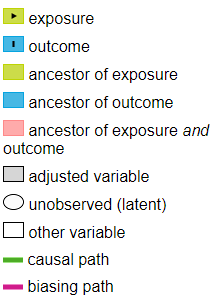 | causal path |
| --- | --- | --- | --- | --- | --- | --- | --- |
| 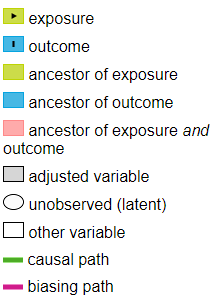 | outcome | 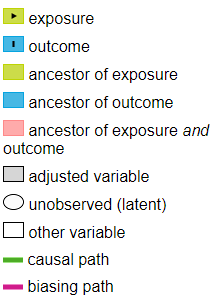 | ancestor of exposure and outcome |  |  | 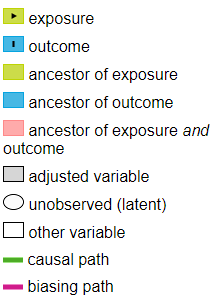 | biasing path |


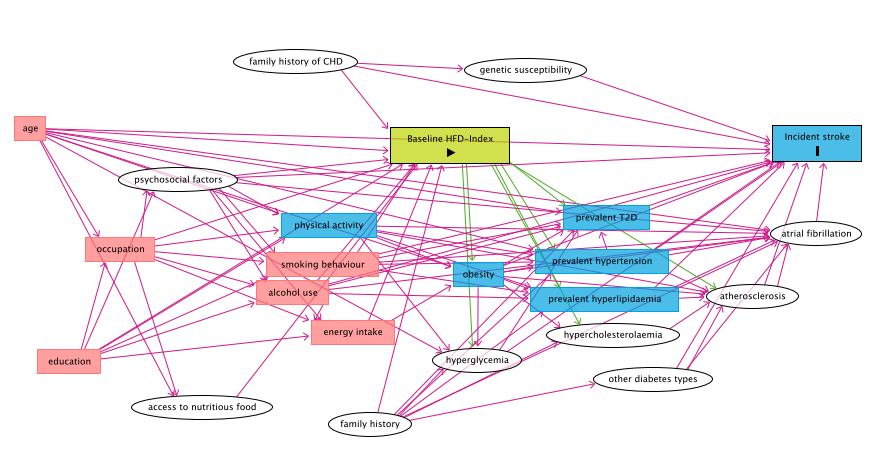


**Supplementary Figure S10.** Directed acyclic graph for the potential causal relationship between baseline Healthy Food Diversity (HFD)-Index and incident stroke.

T2D = type 2 diabetes, CHD = coronary heart disease.

Legend:

| 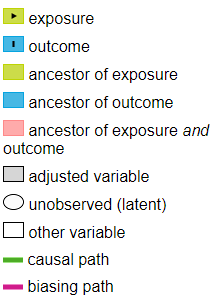 | exposure | 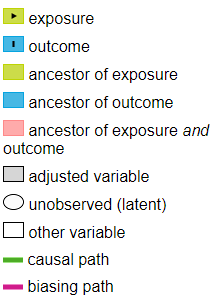 | ancestor of outcome | 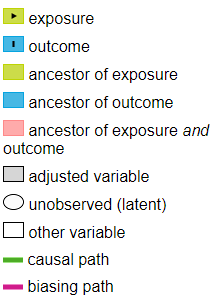 | unobserved | 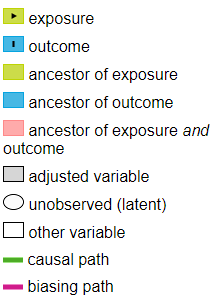 | causal path |
| --- | --- | --- | --- | --- | --- | --- | --- |
| 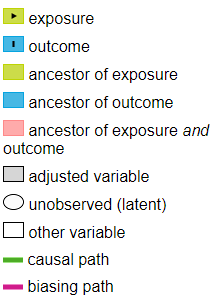 | outcome | 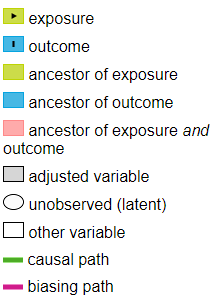 | ancestor of exposure and outcome |  |  | 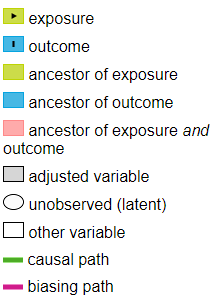 | biasing path |

## Supplementary Methods “Calculation of the HFD-Index”

The diversity component of the HFD-Index is based on the Berry-Index, which is defined as equation 1 and ranges between 0, indicating the consumption of only one food item, and nearly 1 according to the increasing number of foods^1^.

$Berry-Index=1-\sqrt{s_{i}^{2}}$ equation 1^1^

$s_{i}$ = share of each individual food item $i$ by weight of total food intake

The Berry-Index was modified by incorporating a Health Value, which considers the quality of the total food intake according to the dietary guidelines of the German Nutrition Society (DGE)^1,2^. To define the healthiness of the individual diet, Drescher *et al.* created health factors for each food group based on the recommended proportion in the diet in concordance with the German nutrition circle and the German food pyramid illustrating quantitative and qualitative aspects of the DGE dietary guidelines^1,3^. The nutrition circle presents the recommended proportion of six food groups, which were further aggregated into three main food groups “plant foods”, “animal foods”, and “fats and oils”. The recommended proportions of these three food groups describe their Health Value, which are 0.73, 0.25 and 0.02 for “plant foods”, “animal foods”, and “fats and oils”, respectively^1^. The qualitative dimension within these three main food groups is illustrated by the German food pyramid hierarchising food subgroups based on its recommended distribution in the diet, which was further quantified by Drescher *et al.*^1,4^. The proportion of each food subgroup within the main food group was calculated and represented the Health Value of the food subgroups^1^. The proportions or Health Value of the main food groups were multiplied by the proportions or Health Value of the food subgroups to yield the health factor for the fifteen food subgroups (Supplementary Table S9)^1^. Based on the proposed definition^1^, the according food items collected within this study were comprised into three main food groups and fifteen food subgroups (Supplementary Table S10). The share of each individual food item by weight was multiplied by its corresponding established health factor and summed up to calculate the individual Health Value (equation 2) ^1^.

$Health Value = \sum{hf}_{i} \cdot s_{i}$ equation 2

${hf}_{i}$ = health factor of each individual food item $i$

$s_{i}$ = share of each individual food item $i$ by weight of total food intake

To ensure that the Health Value ranges between 1 and nearly 0, it was divided by its maximum 0.26. The combination of the Berry-Index and the Health Value resulted in the final HFD-Index using equation 3.

$HFD-Index =\left( 1-\sqrt{s_{i}^{2}} \right)\cdot(\sum{hf}_{i} \cdot s_{i}$) equation 3

$s_{i}$ = share of each individual food item $i$ by weight of total food intake

${hf}_{i}$ = health factor of each individual food item $i$

**Supplementary Table S1.** Descriptive intake of energy-adjusted food groups across the tertiles of the Healthy Food Diversity (HFD)-Index among 10,551 men and 16,080 women from the EPIC-Potsdam study population at baseline.

|  |  | **Men** | | | | | **Women** | | |
| --- | --- | --- | --- | --- | --- | --- | --- | --- | --- |
| **Characteristic** | **Total** | **Tertile 1** | | **Tertile 2** | **Tertile 3** | | **Tertile 1** | **Tertile 2** | **Tertile 3** |
| HFD-Index (range) | 0.10 - 0.89 | 0.10 - 0.42 | 0.42 - 0.50 | | | 0.50 - 0.76 | 0.10 - 0.48 | 0.48 - 0.56 | 0.56 - 0.89 |
| Number | 26,591 | 3,502 | 3,504 | | | 3,505 | 5,359 | 5,361 | 5,360 |
| **Food group (g/day per 1000 kcal)** |  |  | |  |  | |  |  |  |
| Vegetables and fruits | 208 (144-293) | 102 (79-127) | | 159 (133-190) | 257 (211-330) | | 155 (122-188) | 238 (205-278) | 359 (302-435) |
| Wholemeal products | 12 (3-33) | 3 (0-12) | | 8 (2-23) | 15 (4-40) | | 9 (3-24) | 17 (5-38) | 26 (9-52) |
| Potatoes | 37 (23-55) | 33 (21-51) | | 39 (25-57) | 39 (24-57) | | 34 (20-52) | 38 (24-56) | 36 (22-55) |
| White-meal products | 71 (50-94) | 82 (59-106) | | 79 (58-102) | 71 (50-93) | | 72 (51-96) | 69 (50-91) | 60 (41-81) |
| Snacks and sweets | 77 (53-108) | 100 (65-161) | | 78 (55-109) | 66 (46-90) | | 91 (62-130) | 78 (55-104) | 64 (45-85) |
| Fish / low-fat meat (products) | 33 (10-57) | 13 (6-46) | | 34 (10-63) | 43 (12-66) | | 24 (8-50) | 37 (12-57) | 40 (18-57) |
| Low-fat milk / dairy | 13 (0-67) | 0 (0-36) | | 6 (0-45) | 10 (0-46) | | 14 (0-94) | 22 (0-83) | 26 (1-75) |
| Milk / dairy (products) | 43 (20-84) | 40 (19-82) | | 37 (18-70) | 30 (14-56) | | 61 (27-122) | 51 (24-95) | 39 (18-73) |
| Meat products / eggs | 17 (9-53) | 49 (14-72) | | 23 (9-63) | 14 (8-50) | | 24 (10-55) | 16 (8-45) | 12 (7-27) |
| Bacon | 4 (2-8) | 4 (2-8) | | 4 (2-8) | 4 (2-7) | | 4 (1-8) | 4 (2-8) | 4 (2-7) |
| Oilseed rape / walnut oil | 0.0 (0.0-0.0) | 0.0 (0.0-0.0) | | 0.0 (0.0-0.0) | 0.0 (0.0-0.0) | | 0.0 (0.0-0.0) | 0.0 (0.0-0.0) | 0.0 (0.0-0.0) |
| Wheat germ / soybean oil | 0.2 (0.0-0.8) | 0.1 (0.0-0.3) | | 0.1 (0.0-0.6) | 0.2 (0.0-0.8) | | 0.1 (0.0-0.6) | 0.2 (0.0-1.0) | 0.4 (0.0-1.4) |
| Corn / sunflower oil | 0.7 (0.2-1.4) | 0.4 (0.1-0.8) | | 0.6 (0.2-1.1) | 0.7 (0.3-1.3) | | 0.6 (0.2-1.3) | 0.9 (0.3-1.7) | 1.1 (0.4-2.2) |
| Margarines / butter | 11 (7-15) | 11 (8-16) | | 11 (7-15) | 10 (7-14) | | 11 (7-16) | 11 (7-15) | 10 (6-14) |
| Lard / vegetable fat | 0.4 (0.1-0.9) | 0.2 (0.1-0.6) | | 0.3 (0.1-0.7) | 0.3 (0.1-0.8) | | 0.4 (0.1-0.8) | 0.4 (0.1-1.0) | 0.5 (0.1-1.1) |

Data are shown as median (interquartile range) unless otherwise stated.

**Supplementary Table S2.** Energy-adjusted Spearman correlation coefficients between the Healthy Food Diversity (HFD)-Index, Berry-Index, Health Value and intake of macro- and micronutrients, dietary fibre, cholesterol and other diet quality scores in men and women from the EPIC-Potsdam study population.

|  | **Men** | | | **Women** | | |
| --- | --- | --- | --- | --- | --- | --- |
| Number | 10,511 | | | 16,080 | | |
|  | **HFD-Index** | **Berry-Index** | **Health Value** | **HFD-Index** | **Berry-Index** | **Health Value** |
| HFD-Index | 1.00 | 0.19 | 0.99 | 1.00 | 0.10 | 0.98 |
| Berry-Index | 0.19 | 1.00 | 0.07 | 0.10 | 1.00 | -0.03 |
| Health Value | 0.99 | 0.07 | 1.00 | 0.98 | -0.03 | 1.00 |
| **Nutrients** |  |  |  |  |  |  |
| Total protein | -0.07 | 0.07 | -0.08 | -0.14 | 0.08 | -0.16 |
| Total fat | -0.22 | 0.25 | -0.26 | -0.28 | 0.29 | -0.32 |
| SFA | -0.28 | 0.19 | -0.31 | -0.35 | 0.18 | -0.38 |
| MUFA | -0.21 | 0.27 | -0.25 | -0.25 | 0.33 | -0.30 |
| PUFA | 0.03 | 0.17 | 0.01 | 0.02 | 0.22 | -0.01 |
| Cholesterol | -0.22 | 0.24 | -0.26 | -0.26 | 0.25 | -0.30 |
| Total carbohydrates | 0.17 | -0.26 | 0.20 | 0.22 | -0.28 | 0.26 |
| Disaccharides | 0.07 | -0.10 | 0.08 | 0.03 | -0.23 | 0.07 |
| Dietary fibre | 0.34 | 0.00 | 0.34 | 0.37 | 0.07 | 0.35 |
| Calcium | 0.01 | -0.12 | 0.03 | -0.07 | -0.20 | -0.03 |
| Magnesium | 0.31 | -0.11 | 0.33 | 0.31 | -0.14 | 0.33 |
| Vitamin A | 0.01 | 0.13 | -0.01 | 0.05 | 0.19 | 0.02 |
| Total folate | 0.43 | -0.06 | 0.45 | 0.45 | -0.05 | 0.46 |
| Vitamin C | 0.72 | 0.03 | 0.73 | 0.73 | -0.04 | 0.74 |
| Vitamin E | 0.32 | 0.20 | 0.29 | 0.36 | 0.23 | 0.33 |
| **Other diet quality scores** |  |  |  |  |  |  |
| DASH | 0.62 | 0.16 | 0.61 | 0.22 | 0.18 | 0.20 |
| MedPyr | 0.22 | 0.21 | 0.20 | 0.25 | 0.22 | 0.22 |

SFA = saturated fatty acids; MUFA = mono-unsaturated fatty acids; PUFA = poly-unsaturated fatty acids; DASH = Dietary Approaches to Stop Hypertension; MedPyr = Mediterranean Pyramid.

**Supplementary Table S3.** Modification of the effect of the Healthy Food Diversity (HFD)-Index on the incidence of type 2 diabetes, myocardial infarction and stroke by sex in the EPIC-Potsdam study population.

|  | **Low HFD** | | **High HFD** | | **High HFD vs. low HFD within strata of sex** |
| --- | --- | --- | --- | --- | --- |
|  | Cases,  n/person-years | HR (95% CI) | Cases,  n/person-years | HR (95% CI) | HR (95% CI) |
| **Type 2 diabetes** |  |  |  |  |  |
| Women | 363/82,700 | Ref. | 321/80,973 | 0.95 (0.82, 1.11) | 0.94 (0.80, 1.09) |
| Men | 417/50,719 | 1.77 (1.50, 2.09) | 436/47,962 | 1.84 (1.56, 2.16) | 1.04 (0.91, 1.20) |
| **RERI (95% CI) =** **0.11 (-0.17, 0.39)** | | | | | |
| **Myocardial infarction** |  |  |  |  |  |
| Women | 60/87,315 | Ref. | 55/85,504 | 0.91 (0.63, 1.32) | 0.96 (0.66, 1.40) |
| Men | 134/54,130 | 2.31 (1.63, 3.27) | 127/51,875 | 2.25 (1.60, 3.18) | 0.97 (0.75, 1.24) |
| **RERI (95% CI) = 0.03 (-0.63, 0.69)** | | | | | |
| **Stroke** |  |  |  |  |  |
| Women | 76/86,980 | Ref. | 99/85,078 | 1.24 (0.92, 1.68) | 1.28 (0.94, 1.73) |
| Men | 142/55,134 | 2.34 (1.69, 3.24) | 95/53,720 | 1.40 (1.00, 1.97) | 0.59 (0.45, 0.77) |
| **RERI (95% CI) = -1.18 (-1.93, -0.43)** | | | | | |

Hazard Ratios (HR) and 95% confidence intervals (CI) were calculated by Cox proportional hazards regression across HFD-Index categories based on sex-specific median HFD-Index (men: HFD > 0.46 = high HFD, HFD ≤ 0.46 = low HFD; women: HFD > 0.52 = high HFD, HFD ≤ 0.52 = low HFD).

Models were adjusted for age, education, occupation, smoking status, alcohol intake, total energy intake, physical activity, prevalent hypertension, body mass index, residuals of waist circumference regressed on body mass index, and for vitamin supplementation (only type 2 diabetes) or prevalent type 2 diabetes and hyperlipidaemia (only myocardial infarction and stroke). Ref. = reference group, RERI = relative excess risk due to interaction.

**Supplementary Table S4.** Modification of the effect of the Healthy Food Diversity (HFD)-Index on the incidence of stroke by age in women from the EPIC-Potsdam study population.

|  | **Low HFD** | | **High HFD** | | **High HFD vs. low HFD within strata of age** |
| --- | --- | --- | --- | --- | --- |
|  | Cases,  n/person-years | HR (95% CI) | Cases,  n/person-years | HR (95% CI) | HR (95% CI) |
| **Stroke** |  |  |  |  |  |
| Women < 51 years old | 22/50,332 | Ref. | 26/46,803 | 1.27 (0.72, 2.24) | 1.37 (0.81, 2.32) |
| Women ≥ 51 years old | 54/36,647 | 0.67 (0.31, 1.48) | 73/38,275 | 0.86 (0.40, 1.86) | 1.19 (0.85, 1.66) |
| **RERI (95% CI) = -0.08 (-0.81, 0.64)** | | | | | |

Hazard Ratios (HR) and 95% confidence intervals (CI) were calculated by Cox proportional hazards regression across HFD-Index categories based on sex-specific median HFD-Index (HFD > 0.52 = high HFD, HFD ≤ 0.52 = low HFD).

Models were adjusted for age, education, occupation, smoking status, alcohol intake, total energy intake, physical activity, prevalent hypertension, prevalent type 2 diabetes, hyperlipidaemia, body mass index, residuals of waist circumference regressed on body mass index. Ref. = reference group, RERI = relative excess risk due to interaction.

**Supplementary Table S5.** Longitudinal association between adherence to the Healthy Food Diversity (HFD)-Index and the incidence of type 2 diabetes (T2D), myocardial infarction (MI) and stroke among men and women from the EPIC-Potsdam study population after exclusion of outcome-specific cases with follow-up time < 2 years.

|  | **Hazard ratio (95% confidence interval)** | | | |
| --- | --- | --- | --- | --- |
|  | **Tertile 1** | **Tertile 2** | **Tertile 3** | **Per 1 SD** |
| **Type 2 diabetes*** |  | | |  |
| **Men** (N = 9,552) |  |  |  |  |
| Cases, n/person-years | 240/33,463 | 217/32,947 | 253/32,126 | 710/98,536 |
| Model 1 | Ref. | 0.86 (0.71, 1.03) | 1.01 (0.84, 1.20) | 1.01 (0.94, 1.10) |
| Model 2 | Ref. | 0.90 (0.75, 1.08) | 1.07 (0.89, 1.28) | 1.04 (0.96, 1.13) |
| Model 3 | Ref. | 0.90 (0.75, 1.09) | 1.01 (0.84, 1.21) | 1.02 (0.94, 1.10) |
| **Women** (N = 15,268) |  |  |  |  |
| Cases, n/person-years | 204/54,722 | 198/54,460 | 182/54,376 | 584/163,557 |
| Model 1 | Ref. | 0.91 (0.75, 1.11) | 0.84 (0.68, 1.02) | 0.90 (0.83, 0.98) |
| Model 2 | Ref. | 0.94 (0.77, 1.14) | 0.86 (0.70, 1.05) | 0.92 (0.84, 1.00) |
| Model 3 | Ref. | 0.94 (0.77, 1.14) | 0.89 (0.73, 1.09) | 0.94 (0.86, 1.03) |
| **Myocardial infarction°** |  | | |  |
| **Men** (N = 9,986) |  |  |  |  |
| Cases, n/person-years | 78/35,521 | 70/35,706 | 63/34,729 | 211/105,956 |
| Model 1 | Ref. | 0.82 (0.59, 1.13) | 0.74 (0.53, 1.04) | 0.91 (0.79, 1.05) |
| Model 2 | Ref. | 0.89 (0.64, 1.24) | 0.83 (0.59, 1.17) | 0.97 (0.84, 1.12) |
| Model 3 | Ref. | 0.88 (0.63, 1.23) | 0.79 (0.56, 1.12) | 0.95 (0.82, 1.10) |
| **Women** (N = 15,963) |  |  |  |  |
| Cases, n/person-years | 30/57,461 | 40/57,861 | 35/57,485 | 105/172,807 |
| Model 1 | Ref. | 1.22 (0.76, 1.96) | 1.07 (0.66, 1.75) | non-linearity |
| Model 2 | Ref. | 1.39 (0.86, 2.25) | 1.24 (0.75, 2.04) |  |
| Model 3 | Ref. | 1.37 (0.85, 2.22) | 1.21 (0.74, 2.00) |  |
| **Stroke^#^** |  |  |  |  |
| **Men** (N=10,311) |  |  |  |  |
| Cases, n/person-years | 78/36,189 | 85/36,443 | 51/36,198 | 214/108,830 |
| Model 1 | Ref. | 0.95 (0.70, 1.29) | 0.57 (0.40, 0.81) | 0.83 (0.72, 0.96) |
| Model 2 | Ref. | 0.97 (0.71, 1.32) | 0.56 (0.39, 0.81) | 0.83 (0.72, 0.96) |
| Model 3 | Ref. | 0.93 (0.68, 1.28) | 0.52 (0.36, 0.75) | 0.80 (0.69, 0.93) |
| **Women** (N=15,909) |  |  |  |  |
| Cases, n/person-years | 36/57,318 | 60/57,497 | 59/57,220 | 155/172,035 |
| Model 1 | Ref. | 1.54 (1.02, 2.33) | 1.52 (1.00, 2.30) | 1.20 (1.01, 1.44) |
| Model 2 | Ref. | 1.58 (1.04, 2.40) | 1.55 (1.02, 2.36) | 1.22 (1.02, 1.45) |
| Model 3 | Ref. | 1.57 (1.03, 2.38) | 1.53 (1.00, 2.33) | 1.22 (1.02, 1.46) |

Hazard Ratios (HR) and 95% confidence intervals (CI) were calculated by Cox proportional hazards regression across the tertiles of the HFD-Index and per 1 standard deviation (SD) of the HFD-Index.

Ref. = reference group.

Model 1: adjusted for age; Model 2: Model 1 + education, occupation, smoking status, alcohol intake, total energy intake, physical activity; Model 3: Model 2 + prevalent hypertension, body mass index, residuals of waist circumference regressed on body mass index, and vitamin supplementation (only T2D) or prevalent type 2 diabetes and hyperlipidaemia (only MI and stroke).

Exclusion of incident *T2D cases, °T2D and MI cases, ^#^T2D and stroke cases with follow-up time < 2 years.

**Supplementary Table S6.** Longitudinal association between adherence to the Berry-Index (BI), the Health Value (HV) and the Healthy Food Diversity (HFD)-Index, and the incidence of type 2 diabetes, myocardial infarction and stroke among men and women from the EPIC-Potsdam study population.

|  | **Hazard ratio (95% confidence interval)** | | |
| --- | --- | --- | --- |
|  | **Per 1 SD (BI)** | **Per 1 SD (HV)** | **Per 1 SD (HFD)** |
| **Type 2 diabetes** |  |  |  |
| **Men** (N=9,695) |  |  |  |
| Cases, n/person-years | 853/98,681 | 853/98,681 | 853/98,681 |
| Model 1 | 0.89 (0.85, 0.95) | 1.05 (0.98, 1.13) | 1.01 (0.94, 1.09) |
| Model 2 | 0.92 (0.87, 0.97) | 1.07 (1.00, 1.15) | 1.04 (0.97, 1.12) |
| Model 3 | 0.94 (0.88, 0.99) | 1.05 (0.98, 1.12) | 1.02 (0.95, 1.10) |
| **Women** (N=15,368) |  |  |  |
| Cases, n/person-years | 684/16,3672 | 684/16,3672 | 684/16,3672 |
| Model 1 | 0.96 (0.89, 1.03) | 0.92 (0.85, 1.00) | 0.92 (0.85, 1.00) |
| Model 2 | 0.98 (0.91, 1.06) | 0.94 (0.87, 1.02) | 0.94 (0.87, 1.02) |
| Model 3 | 0.99 (0.91, 1.07) | 0.97 (0.89, 1.05) | 0.97 (0.89, 1.05) |
| **Myocardial infarction** |  |  |  |
| **Men** (N = 10,037) |  |  |  |
| Cases, n/person-years | 261/106,005 | 261/106,005 | 261/106,005 |
| Model 1 | 0.89 (0.81, 0.98) | 0.99 (0.87, 1.13) | 0.96 (0.84, 1.09) |
| Model 2 | 0.92 (0.84, 1.02) | 1.06 (0.93, 1.20) | 1.02 (0.90, 1.17) |
| Model 3 | 0.93 (0.85, 1.03) | 1.03 (0.91, 1.17) | 1.00 (0.88, 1.14) |
| **Women** (N = 15,974) |  |  |  |
| Cases, n/person-years | 115/172,820 | 115/172,820 | 115/172,820 |
| Model 1 | 0.95 (0.80, 1.13) | 0.94 (0.77, 1.15) | non-linearity |
| Model 2 | 1.05 (0.87, 1.27) | 1.00 (0.82, 1.21) |  |
| Model 3 | 1.05 (0.87, 1.27) | 0.99 (0.82, 1.21) |  |
| **Stroke** |  |  |  |
| **Men** (N=10,335) |  |  |  |
| Cases, n/person-years | 237/108,854 | 237/108,854 | 237/108,854 |
| Model 1 | 0.93 (0.83, 1.04) | 0.83 (0.72, 0.96) | 0.83 (0.72, 0.95) |
| Model 2 | 0.97 (0.86, 1.09) | 0.84 (0.73, 0.97) | 0.83 (0.72, 0.95) |
| Model 3 | 0.98 (0.87, 1.10) | 0.80 (0.69, 0.93) | 0.80 (0.70, 0.92) |
| **Women** (N=15,930) |  |  |  |
| Cases, n/person-years | 175/172,058 | 175/172,058 | 175/172,058 |
| Model 1 | 1.00 (0.86, 1.18) | 1.18 (1.00, 1.38) | 1.18 (1.00, 1.39) |
| Model 2 | 1.03 (0.87, 1.21) | 1.20 (1.02, 1.41) | 1.20 (1.01, 1.41) |
| Model 3 | 1.03 (0.87, 1.22) | 1.20 (1.02, 1.41) | 1.20 (1.01, 1.42) |

Hazard Ratios (HR) and 95% confidence intervals (CI) were calculated by Cox proportional hazards regression per 1 standard deviation (SD) of the HFD-Index and its components BI and HV.

Model 1: adjusted for age; Model 2: Model 1 + education, occupation, smoking status, alcohol intake, total energy intake, physical activity; Model 3: Model 2 + prevalent hypertension, body mass index, residuals of waist circumference regressed on body mass index, and BI (only HV) or HV (only BI), and vitamin supplementation (only type 2 diabetes) or prevalent type 2 diabetes and hyperlipidaemia (only myocardial infarction and stroke).

**Supplementary Table S7.** Longitudinal age-stratified association between adherence to the Healthy Food Diversity (HFD)-Index and the incidence of stroke among women from the EPIC-Potsdam study population.

|  | **Hazard ratio (95% confidence interval)** | |
| --- | --- | --- |
|  | **Per 1 SD HFD** | |
| **Stroke** |  |  |
|  | **Women < 51 years old** (N=8909) | **Women ≥ 51 years old** (N=7021) |
| Cases, n/person-years | 48/97,135 | 127/74,923 |
| Model 1 | 1.06 (0.79, 1.43) | 1.23 (1.01, 1.50) |
| Model 2 | 1.12 (0.83, 1.51) | 1.24 (1.01, 1.51) |
| Model 3 | 1.12 (0.83, 1.52) | 1.24 (1.01, 1.51) |

Hazard Ratios (HR) and 95% confidence intervals (CI) were calculated by Cox proportional hazards regression per 1 standard deviation (SD) of the HFD-Index.

Model 1: crude; Model 2: Model 1 + adjusted for education, occupation, smoking status, alcohol intake, total energy intake, physical activity; Model 3: Model 2 + prevalent hypertension, prevalent type 2 diabetes, hyperlipidaemia, body mass index, residuals of waist circumference regressed on body mass index.

**Supplementary Table S8.** Selected covariate data collected in the EPIC-Potsdam study at baseline.

|  | **Categories or Unit** |
| --- | --- |
| **Basic information** |  |
| Age | - continuous in years |
| Sex | - male - female |
| **Sociodemographic information** |  |
| Education | - no vocational training - technical college - university |
| Occupation | - full-time (≥ 35 hours / week) - part-time (15 - < 35 hours / week) - hourly (< 15 hours / week) - jobless or retraining - (early) retirement or invalidity pension - unemployed |
| **Lifestyle information** |  |
| Smoking status | - never smoker - ex-smoker - smoker |
| Alcohol lifetime pattern | - never - former, never heavy (≤ 30 / 60) - former, periodically heavy - current, always light (≤ 1 / 2) - current, always below the limit (≤ 12 / 24) - current, never heavy (≤ 30 / 60) - current, periodically heavy - current, always heavy (> 30 / 60) |
| Total energy intake | - continuous in kcal / day - calculated from food frequency questionnaire |
| Total sports | - continuous in hours / week - summation of sport, cycling and gardening activities |
| Vitamin supplementation | - yes - no |
| Waist circumference | - continuous (cm) - adjusted for body mass index (continuous, kg/m²) using the residual method |
| Prevalent hypertension | - yes - no |
| Prevalent type 2 diabetes | - yes - no |
| Prevalent hyperlipidaemia | - yes - no |

**Supplementary Table S9.** Health factors for 15 food subgroups of the Healthy Food Diversity-Index developed by Drescher *et al.*^2^.

| **Food group** | **Share of food subgroup, %** | **Health factors** |
| --- | --- | --- |
| **Plant foods (73%)** |  | **0.73 x** |
| Vegetables / fruits / leaf salads / juices* | 36 | 0.36 = 0.2628 |
| Wholemeal products / paddy | 28 | 0.28 = 0.2044 |
| Potatoes | 20 | 0.20 =0.1460 |
| White-meal products / peeled rice | 12 | 0.12 = 0.0876 |
| Snacks and sweets | 4 | 0.04 = 0.0292 |
| **Animal foods (25%)** |  | **0.25 x** |
| Fish / low-fat meat / low-fat meat products | 36 | 0.36 = 0.090 |
| Low-fat milk / low-fat dairy products | 28 | 0.28 = 0.070 |
| Milk / dairy products | 20 | 0.20 = 0.050 |
| Meat products / sausages / eggs | 12 | 0.12 = 0.030 |
| Bacon | 4 | 0.04 = 0.010 |
| **Fats and oils (2%)** |  | **0.02 x** |
| Oilseed rape / walnut oil | 36 | 0.36 = 0.0072 |
| Wheat germ oil / soybean oil | 28 | 0.28 = 0.0056 |
| Corn oil / sunflower oil | 20 | 0.20 = 0.0040 |
| Margarines / butter | 12 | 0.12 = 0.0024 |
| Lard / vegetable fat | 4 | 0.04 = 0.0008 |

**Supplementary Table S10.** The 15 food subgroups of the Healthy Food Diversity-Index and according food items / groups of the EPIC-Potsdam food frequency questionnaire.

| **Food subgroup (corresponding health factor)** | | |
| --- | --- | --- |
| **Vegetables / fruits / leaf salads / juices (0.2628)** | | |
| - fresh fruits - fresh vegetables - cooked vegetables - tomato sauce - mushrooms | - sauerkraut - gherkin, mixed pickles - coleslaw - nuts | - legumes - fresh fruit juice (100%) - vegetarian dishes |
| **Wholemeal products / paddy (0.2044)** | | |
| - wholegrain bread | - dark bread | - wholegrain rolls |
| **Potatoes (0.146)** | | |
| - cooked/mashed potatoes | - potato / bread dumpling | - potato salad |
| **White-meal products / peeled rice (0.0876)** | | |
| - brown / rye bread - white bread / rolls - toast | - crispbread / croissant / pretzel - cornflakes / crisps | - cereal / grain / muesli - rice - pasta |
| **Snacks and sweets (0.0292)** | | |
| - chips / peanut curls / salt sticks - pizza - quiche / onion tart / bacon tart - fried potatoes - pommes frites / croquette - potato fritter - ketchup - dessert - sweet dishes - canned fruits | - tart / pie - cake without filling - yeast cake and pastry - Danish pastry - sponge cake / cake with cream / custard filling - cookies - pancake - chocolate - pralines - sweet snack | - ice cream - sugar - honey - marmalade / jam / jelly - hazelnut chocolate spread - lemonade (normal) - coke (normal) - non-alcoholic beer |
| **Fish/low-fat meat/low-fat meat products (0.09)** | | |
| - fish (filet, natural or breaded) - fish (canned, smoked) - lean beef (steak, filet / roasted, cooked / goulash, meat strips / roulade) - lean veal / lamb / rabbit - lean black pudding | - lean pork (schnitzel, chop / roasted / goulash, meat strips / cooked / *kasseler*, cured pork chop / belly) - lean broiler - lean turkey (meat strips, schnitzel) - lean minced meat sauce, hash - lean bratwurst | - lean meat balls, hamburger, meat loaf - lean sausages (wiener, frankfurter, bockwurst) - lean ham sausage, *Lyoner*, chausseur sausage / Salami / *Teewurst* |

**Supplementary Table S10.** Continued.

| **Low-fat milk / low-fat dairy products (0.07)** | | |
| --- | --- | --- |
| - milk (≤ 1.5% fat) - milkshake (≤ 1.5% fat) - curd (5% fat) | - (fruit) yogurt (≤ 1.5% fat) - cream cheese (low-fat) | - soft cheese (low-fat) - sliced cheese (low-fat) |
| **Milk / dairy products (0.05)** | | |
| - milk (3.5% or varied fat) - milkshake (3.5% or varied fat) - (fruit) yoghurt (3.5%, 10 % or varied fat) | - curd (20%, 40% or varied fat) - cream cheese (fat or varied fat) - soft cheese (normal or varied fat) | - processed cheese sliced cheese (normal or varied fat) - soured milk, kefir - whipped cream |
| **Meat products, sausages, eggs (0.03)** | | |
| - medium / fat beef (steak, filet / roasted, cooked / goulash, meat strips / roulade) - medium / fat veal / lamb / rabbit - medium / fat black pudding - liver - liver sausage - cooked egg - fried / scrambled eggs, omelette | - medium / fat pork (schnitzel, chop / roasted / goulash, meat strips / cooked / *kasseler*, cured pork chop / belly) - medium/fat broiler - medium/fat turkey (meat strips, schnitzel) - medium/fat minced meat sauce, hash | - medium / fat meat balls, hamburger, meat loaf - medium / fat sausages (wiener, frankfurter, bockwurst) - medium / fat ham sausage, *Lyoner*, chausseur sausage / Salami / *Teewurst* - medium / fat bratwurst |
| **Bacon (0.01)** | | |
| - bacon, *kasseler*, cold meat (lean / medium / fat) | | |
| **Oilseed rape / walnut oil (0.0072)** | | |
| - rape seed oil | | |
| **Wheat germ oil / soybean oil (0.0056)** | | |
| - olive oil | - soy oil | - wheat germ oil |
| **Corn oil / sunflower oil (0.004)** | | |
| - sunflower oil - corn oil | - safflower oil - peanut oil | - sesame oil - linseed oil |
| **Margarines / butter (0.0024)** | | |
| - butter | - margarine |  |
| **Lard / vegetables fat (0.0008)** | | |
| - vegetable fat | - lard - animal fat | - cream, crème fraiche, mayonnaise |

## References of the supplement

1 Drescher, L. S., Thiele, S. & Mensink, G. B. A new index to measure healthy food diversity better reflects a healthy diet than traditional measures. *J Nutr* **137**, 647-651, doi:10.1093/jn/137.3.647 (2007).

2 Deutsche Gesellschaft für Ernährung (DGE), e. V.. 10 guidelines of the German Nutrition Society (DGE) for a wholesome diet. (2017).

3 Vadiveloo, M., Dixon, L. B., Mijanovich, T., Elbel, B. & Parekh, N. Development and evaluation of the US Healthy Food Diversity index. *Br J Nutr* **112**, 1562-1574, doi:10.1017/s0007114514002049 (2014).

4 Oberritter, H., Schäbethal, K., Ruesten, A. v. & Boeing, H. The DGE Nutrition Circle - Presentation and Basis of the Food-Related Recommendations from the German Nutrition Society (DGE). Ernaehrungs Umschau international 60 (**2**): 24-29, DOI 10.4455/eu.2013.004
